# Animal pollination increases stability of crop yield across spatial scales

**DOI:** 10.1111/ele.14069

**Published:** 2022-07-17

**Authors:** Jacob Bishop, Michael P. D. Garratt, Shinichi Nakagawa

**Affiliations:** ^1^ Department of Crop Science, School of Agriculture, Policy and Development University of Reading Berkshire UK; ^2^ Centre for Agri‐Environmental Research, School of Agriculture, Policy and Development University of Reading Berkshire UK; ^3^ Evolution and Ecology Research Centre, School of Biological and Environmental Sciences University of New South Wales Sydney New South Wales Australia

**Keywords:** *Brassica napus*, coefficient of variation, insect pollination, lnCVR, *Malus domestica*, meta‐analysis, *Vicia faba*

## Abstract

The benefits of animal pollination to crop yield are well known. In contrast, the effects of animal pollination on the spatial or temporal stability (the opposite of variability) of crop yield remain poorly understood. We use meta‐analysis to combine variability information from 215 experimental comparisons between animal‐pollinated and wind‐ or self‐pollinated control plants in apple, oilseed rape and faba bean. Animal pollination increased yield stability (by an average of 32% per unit of yield) at between‐flower, ‐plant, ‐plot and ‐field scales. Evidence suggests this occurs because yield benefits of animal pollination become progressively constrained closer to the maximum potential yield in a given context, causing clustering. The increase in yield stability with animal pollination is greatest when yield benefits of animal pollination are greatest, indicating that managing crop pollination to increase yield also increases yield stability. These additional pollination benefits have not yet been included in economic assessments but provide further justification for policies to protect pollinators.

## Graphical abstract

We use meta‐analysis to quantify the effect of animal pollination on crop yield stability, combining 215 experimental comparisons between animal pollinated and control plants in apple, oilseed rape and faba bean. Animal pollination increases yield stability (by an average 32%) at between‐flower, ‐plant, ‐plot and ‐field scales. We suggest this occurs because yield benefits of animal pollination become constrained, resulting in clustering at the maximum potential yield in a given context.

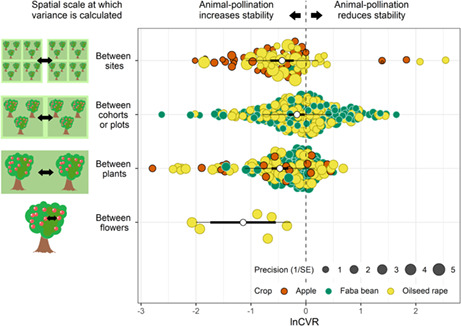

## INTRODUCTION

Crop pollination by animals increases yield in three‐quarters of the world's crop types (Klein et al., 2007). Many empirical studies have been conducted to quantify the extent of these yield benefits and their economic value (Breeze et al., 2016). Research syntheses have estimated that animal pollination accounts for 3–8% of global food production (Aizen et al., 2009) and is worth $235–577 billion globally each year (Potts et al., 2016). Recognition of these benefits has resulted in widespread recommendations to support pollinator populations, and to manage crops in ways that increase pollinator activity (Dicks et al., 2016; Kleijn et al., 2019). In contrast, there is very limited knowledge about how animal pollination affects the stability of crop yield. Crop yield stability across time (low variability in crop yield between years; Knapp & van der Heijden, 2018) and across space (low variability between flowers, plants, fields and/or farms; Maestrini & Basso, 2018) is of comparable importance to agricultural producers as mean yield (Barah et al. 1981; Eskridge, 1990). Increasing the stability of crop yield at a range of spatial scales is necessary to reduce input costs and the environmental impacts of agriculture, close yield gaps and improve yields (Maestrini & Basso, 2018). Research is needed to understand how animal pollination affects crop yield stability to facilitate the management of pollination services to deliver desired yield and yield stability outcomes.

It is intuitive that animal pollination of crops will enhance yield stability at small spatial scales, for example between flowers on a plant or between plants in a field. The majority (c. 70%) of crops that benefit from animal pollination have an intermediate level of pollination dependence (Klein et al., 2007), where yield is increased by animal pollination but some yield is produced by wind or autogamy (hereafter auto‐pollination). In these crops, some ovules, flowers or plants produce seeds by auto‐pollination while others do not (Damgaard et al., 1992; Goodwillie et al., 2005). Enhancing pollination services should increase yield stability at between‐flower or between‐plant scales by facilitating more uniform seed set.

At larger spatial scales (e.g. between areas in a heterogeneous field or between fields) it is less clear how animal pollination might affect yield stability. Alongside animal pollination, crop yield per unit area is influenced by many other factors (e.g. soil fertility and pests; Garibaldi et al., 2018; Tamburini et al., 2019). An increase in ovule fertilisation due to animal pollination should have a greater impact on crop yield if plants have more resources (see multiple limitation hypothesis; Garibaldi et al., 2018). This positive interaction (synergism) of animal pollination with other yield‐supporting factors is commonly observed in experiments (Tamburini et al., 2019) and implies that animal pollination services could exaggerate existing differences in yield (caused, e.g. by differences in nutrient availability between fields or differences in weather between years; see Garratt, Bishop, et al., 2018) and reduce yield stability across space and time. In other cases, animal pollination may compensate for yield‐limiting factors, like heat stress (Bishop et al., 2016) or pest damage (Bartomeus et al., 2015), or may act independently (Garibaldi et al., 2018; Tamburini et al., 2019), each with contrasting outcomes for yield stability. We need an understanding of how animal pollination can be managed alongside other agronomic inputs to improve crop yield and crop yield stability in different cropping systems and contexts.

While empirical studies are typically able to conclude whether there are statistically significant mean effects of animal pollination on crop yield (Aizen et al., 2009; Breeze et al., 2016; Potts et al., 2016), stability (as the opposite of variability) is a summary statistic derived from multiple values and far larger sample sizes are therefore required to identify differences (Mills et al., 2021). Consequently, only a small number of publications have measured the effects of animal pollination on yield stability and they have mostly been correlative (but see Hünicken et al., 2021), either testing associations between the stability of pollinator activity and stability of fruit set (Garibaldi, Steffan‐Dewenter, et al., 2011; Lázaro & Alomar, 2019) or comparing yield stability between pollinator‐dependent and ‐independent crops (Garibaldi, Aizen, et al., 2011; Oguro et al., 2019). Garibaldi, Steffan‐Dewenter, et al. (2011) combined data from 29 empirical studies in 21 crops and showed that a decline in the spatial stability (between‐plants or between‐plots within fields) and temporal stability (between‐days in a season) of insect pollinator activity away from natural habitats coincided with a small decline in spatial stability of fruit set (although this was not significant). A different study tested whether between‐year stability of national‐scale yield was associated with pollination dependence in 99 crop species; finding that crops categorised as more pollinator dependent had less stable yield over time (Garibaldi, Aizen, et al., 2011). This result was not replicated in a study focusing on Japan, and the authors cited systematic differences in agricultural management between pollination‐dependent and ‐independent crops (Oguro et al., 2019). A recent empirical study demonstrated that animal pollination increased the between‐plant and between‐year stability of fruit set within 14 orchards of apples and pears relative to auto‐pollinated control plants (Hünicken et al., 2021). There are several knowledge gaps that need to be addressed; (1) how does animal pollination affect crop yield stability across cropping systems, (2) how does any change in crop yield stability relate to the yield benefit due to animal pollination in a given context, (3) do effects of animal pollination on yield stability extend beyond fruit set to more economically relevant yield measures (see Bishop et al., 2020) and (4) do these effects vary between crop types or across temporal and spatial scales.

Here, we use a meta‐analytic approach to explore how animal pollination affects spatial yield stability in three globally important crops at a range of spatial scales. We synthesise the results of 47 publications and 215 controlled experimental comparisons in apple, oilseed rape and faba bean. These experimental comparisons were designed to compare [mean] differences in yield production between plants receiving an animal pollination treatment and auto‐pollinated control plants (Table 1), but these publications also provide information about the variability (e.g. the standard deviation) of yield between the experimental units in each treatment. We use multi‐level meta‐analytic models (Noble et al., 2017) and the log coefficient of variation ratio (lnCVR) to quantify the relative difference in yield stability between experimental units of animal‐pollinated and auto‐pollinated control plants across crop types and contexts (Knapp & van der Heijden, 2018). A negative lnCVR indicates that the yield of animal‐pollinated plants is more stable (it has a lower coefficient of variation between‐flowers, ‐plants, ‐plots or ‐fields) than control plants (Garibaldi, Steffan‐Dewenter, et al., 2011). The coefficient of variation (CV) has been used in previous analyses of animal pollination and crop yield stability (Garibaldi, Aizen, et al., 2011; Garibaldi, Steffan‐Dewenter, et al., 2011; Hünicken et al., 2021) and corrects for expected increases in variance with the mean (Döring et al., 2015; Knapp & van der Heijden, 2018). To understand whether managing animal pollination to deliver increased crop yield also results in greater yield stability, we test the association between lnCVR and the yield benefit of animal pollination (lnRR; the log ratio of (mean) yield between animal‐pollinated and control plants, see Bishop & Nakagawa, 2021). We then use additional analyses to test how lnCVR varies between crop species, and to test whether changes in lnCVR are associated with resource limitation, or the level of pollinator activity. Our approach enables us to test the effect of animal pollination on yield stability while controlling for confounding factors that correlative studies cannot address.

**TABLE 1:** Summary of publications used in analysis, lnCVR and lnRR estimates are a publication‐level mean across the effect sizes used in our analysis. Publications with more than one row presented findings from more than one independent experiment

Publication	Crop	Country	Spatial scale	lnCVR	lnRR	Effect sizes
Riedel and Wort (1960)	Faba bean	UK	Plot	−0.62	0.53	2
Palmer‐Jones and Clinch (1966)	Apple	New Zealand	Plant	−2.17	2.53	2
Palmer‐Jones and Clinch (1967)	Apple	New Zealand	Plant	−0.77	2.77	1
Palmer‐Jones and Clinch (1968)	Apple	New Zealand	Plant	−0.9	2.39	2
Eisikowitch (1981)	Oilseed rape	UK	Plant	−2.32	2.78	4
Mesquida and Renard (1981)	Oilseed rape	France	Plot	−0.21	0.81	10
Mesquida and Renard (1982)	Oilseed rape	France	Plant	−0.54	1.94	16
Dekhuijzen et al. (1988)	Faba bean	Netherlands	Plant	0.13	−0.09	3
Mesquida et al. (1988)	Oilseed rape	France	Plot	−0.21	0.09	15
Mesquida et al. (1990)	Faba bean	France	Plot	0.15	0.66	24
Kołtowski (1996)	Faba bean	Poland	Plot	0.01	0.25	36
Somerville (1999)	Faba bean	Australia	Plot	−0.81	0.25	3
Ladurner et al. (2004)	Apple	Italy	Plant	−0.48	1.08	12
Kołtowski (2005)	Oilseed rape	Poland	Plot	−0.08	0.05	42
Sabbahi et al. (2005)	Oilseed rape	Canada	Site	−0.22	0.56	12
Benachour et al. (2007)	Faba bean	Algeria	Plot	0.02	0.64	5
Aouar‐sadli et al. (2008)	Faba bean	Algeria	Plot	−0.67	0.06	4
Jauker and Wolters (2008)	Oilseed rape	Germany	Plot	−0.4	0.18	2
Durán et al. (2010)	Oilseed rape	Chile	Plot	−1	0.38	3
Khan et al. (2012)	Oilseed rape	Pakistan	Plant	−0.45	0.52	2
Garratt, Evans, et al. (2014)	Apple	UK	Site	−1.1	1.45	4
Shakeel and Inayatulla (2013)	Oilseed rape	Pakistan	Plot	−0.04	0.3	4
Garratt, Breeze, et al. (2014)	Apple	UK	Site	−0.41	1.49	8
Bartomeus et al. (2014)	Faba bean	UK	Site	−0.28	0.57	1
Garratt, Coston, et al. (2014)	Faba bean	UK	Cohort	0.2	0.12	13
Bartomeus et al. (2014)	Oilseed rape	Sweden	Site	−0.03	0.2	1
Witter et al. (2014)	Oilseed rape	Brazil	Plant	−0.52	0.56	6
Garratt, Coston, et al. (2014)	Oilseed rape	UK	Cohort	0.06	0.38	18
Hudewenz et al. (2014)	Oilseed rape	Germany	Plant	−0.52	0.34	24
Mallinger and Gratton (2015)	Apple	USA	Site	−0.84	2.58	6
Marini et al. (2015)	Oilseed rape	Italy	Plot	−0.51	0.17	6
Garratt et al. (2016)	Apple	UK	Site	−0.48	1.22	2
Bishop et al. (2016)	Faba bean	UK	Cohort	−0.36	0.13	40
St‐Martin and Bommarco (2016)	Faba bean	Sweden	Plant	−0.93	0.35	4
Sutter and Albrecht (2016)	Oilseed rape	Switzerland	Plot	−0.28	0.19	6
Campbell et al. (2017)	Apple	UK	Site	−0.17	1.37	4
Bishop et al. (2017)	Faba bean	UK	Cohort	−0.2	0.73	8
			Plant	−0.11	0.41	8
Ouvrard et al. (2017)	Oilseed rape	Belgium	Flower	−1.09	0.33	6
Zou et al. (2017)	Oilseed rape	China	Site	−0.04	0.16	2
Porcel et al. (2018)	Apple	Sweden	Site	−0.97	1.54	1
Kyllönen (2018)	Faba bean	Finland	Plot	0.88	0.54	3
Garratt, Bishop, et al. (2018)	Oilseed rape	UK	Plant	−0.08	0.16	16
			Site	0.5	0.13	6
Garratt, Brown et al. (2018)	Oilseed rape	UK	Site	−0.07	0.01	3
Perrot et al. (2018)	Oilseed rape	France	Site	−0.73	0.45	12
Bishop et al. (2020)	Faba bean	UK	Plant	−0.2	0.24	60
			Plot	0.09	−0.01	18
Toivonen et al. (2019)	Oilseed rape	Finland	Site	−0.32	0.92	2
Hünicken et al. (2020)	Apple	Argentina	Site	0.48	1.33	2
Pérez‐Méndez et al. (2020)	Apple	Argentina	Site	−1.26	1.67	3
Greenop et al. (2020)	Faba bean	UK	Plant	−0.47	1.66	1

## MATERIALS AND METHODS

### Study systems

Our analysis focuses on three major animal pollinated crops, apple (*Malus domestica*), faba bean (*Vicia faba*) and oilseed rape (*Brassica napus*). These crops, and different cultivars within these crops, depend to varying extents on pollination by insects for maximum yield production; apple has great yield dependence on animal pollination (40−<90% yield reduction without animal pollination), while faba bean and oilseed rape have modest dependence (32.9% (21–43%) and 10−<40% respectively; Bishop & Nakagawa, 2021; Klein et al., 2007). The crops are economically important globally, they vary in their biology including mass flowering annual crops and a perennial tree crop, and are some of the most well‐studied crops with regards the role of animal pollination. Therefore, these species make an ideal case study in which to investigate the role of animal pollination in crop yield stability.

### Identifying publications and extracting data

We use several existing systematic reviews about animal pollination benefits to our three study crops (Bishop & Nakagawa, 2021; Ouvrard & Jacquemart, 2019; Pardo & Borges, 2020; Woodcock et al., 2019) as the starting point to identify relevant primary publications for our analysis. We updated each review to October 2020 by searching Web of Science using the same search terms as the original reviews. This approach resulted in a total of 279 unduplicated publications that we screened. We present a PRISMA diagram to illustrate our review process (Figure S1) and provide a PRISMA‐EcoEvo checklist detailing our literature review and other aspects of our meta‐analysis (O'Dea et al., 2021). We used several inclusion criteria to identify which of the publications to include in our analysis; each publication needed to present (1) at least one experimental comparison between plants (or other experimental unit e.g. flower or plot) receiving a hand and/or animal pollination treatment, and an auto‐pollinated control (that was, e.g. enclosed in mesh cage to exclude pollinating insects), (2) separate variability information for animal‐pollinated and auto‐pollinated plants (e.g. standard deviation or a convertible alternative such as standard error), (3) sample size for animal‐pollinated and auto‐pollinated plants, (4) unambiguous information about the spatial or temporal scale at which the mean and variability are calculated. Where the first criterion was met but variability information was not present or the scale and/or number of replicates were ambiguous, we contacted publication authors to request this information for manuscripts with a publication date of 2000 onwards. Several authors provided additional data (see acknowledgements). We manually extracted quantitative data from the publications from tables and supplementary data, or if presented in figures, we used the metaDigitise package (Pick et al., 2019).

Publications reported several different yield responses that have different relationships to economically relevant yield production (Bishop et al., 2020). We measure the stability of a wide range of these response measures together in our analysis including those directly linked to yield (yield mass, seed number, pod number) and to fertilisation (seeds per fruit, percentage fruit set) allowing us to include multiple crops in our analysis. All are positively associated with yield mass (supplementary information). There were no direct (total) yield data available for apple due to the nature of experiments in this crop which focus on flower or branch scale manipulations (Webber et al., 2020). Where initial fruit set and fruit set at harvest were reported, we used only fruit set at harvest as this is most relevant to yield. We did not measure the stability of response measures relating to quality (e.g. apple width, thousand seed weight) in our analyses. When publications reported yield using more than one response measure for each experimental comparison, we included all reported effect sizes in our analysis. We therefore included a total of 498 effect sizes from 215 experimental comparisons and 47 publications in our main analyses. The number of experimental comparisons is larger than the number of publications because publications often compared responses in several crop cultivars, tested different animal pollination treatments (e.g. hand‐pollination, open‐pollinated) or had other experimental treatments. We use random effects and a variance–covariance matrix to address nonindependence between effect sizes (see below).

### Measuring stability

All publications tested for differences in the mean yield response between animal‐pollinated and auto‐pollinated plants and presented the variance (as SD or SE) in yield between the experimental units in each group. We use this information to calculate the log ratio of the coefficient of variation (lnCVR) and the log ratio of the variance (lnVR; absolute stability) between experimental units of animal‐pollinated and auto‐pollinated control plants. These ratio measures allow us to combine all effect sizes and compare the yield variability of animal‐pollinated and control plants within a powerful meta‐analytic framework (Nakagawa et al., 2015). A ratio less than zero indicates that animal‐pollinated plants are more stable (less variable) across space than their auto‐pollinated counterparts. There is a strong mean–variance relationship in our data (Figure S2) and the lnCVR accounts for expected changes in variance associated with changes in the mean and therefore measures the difference in yield stability per unit of yield (Döring et al., 2015). lnVR, is not mean‐corrected and simply compares the ratio of variances, representing absolute stability (see Knapp & van der Heijden, 2018). We use modified versions of lnCVR and lnVR that correct for bias associated with small sample sizes (Senior et al., 2020).

The lnCVR (and the coefficient of variation itself, CV) assumes a linear slope coefficient of 1 between ln(mean) and ln(SD). A different slope between ln(mean) and ln(SD) results in over‐ or under‐correction of the mean (Döring et al., 2015), potentially resulting in erroneous conclusions about stability (Usui et al., 2021). Therefore, we also tested an arm‐based model using ln(SD) as the response and the treatment condition (animal‐pollinated or control) and ln(mean) as fixed effects. In this arm‐based model, the regression coefficient for the treatment condition becomes equivalent to the overall effect size in the model using lnCVR (see Senior et al., 2016). The difference is that in the arm‐based model, the ln(mean) term estimates the relationship between ln(SD) and ln(mean), while in the lnCVR model a 1:1 relationship between ln(SD) and ln(mean) is assumed.

In addition to stability ratios, we also quantify the effect of animal pollination on mean yield (the yield benefit of animal pollination) using a modified version of the log response ratio (lnRR) that corrects for bias associated with small sample sizes (Senior et al., 2020). A positive lnRR indicates that animal pollination increases mean yield relative to auto‐pollinated controls.

### Multi‐level meta‐analysis models

There were several sources of nonindependence (violation of assumption of data independence; Noble et al., 2017) in the data. We used multi‐level meta‐analytic models, which included random effects to estimate and account for the correlation of effect sizes from within the same publication and from the same experimental comparison. Regarding the latter, publications often reported multiple yield responses (e.g. the mass of seeds, the number of seeds) and we assumed that effect sizes measured on the same experimental units were correlated but that their sampling errors were not (Noble et al., 2017). We tested the inclusion of a random effect for cultivar; 11 of the publications tested responses to animal pollination in more than one cultivar and eight cultivars were tested in more than one publication, but this random effect did not improve model performance (see supplementary information). We also included a random effect for each effect size (data row) which is necessary to estimate the residual (unexplained) heterogeneity.

Several publications included comparisons of more than one animal pollination treatment to a single control group. These effect sizes are therefore not independent from one another. For the lnCVR, lnVR and lnRR analyses, we accounted for shared controls using a variance–covariance matrix which specifically modelled the correlation of sampling errors (Senior et al., 2020) arising due the repeated comparisons to the same control group. For the arm‐based ln(SD) analyses, we instead used robust variance estimation (Hedges et al., 2010; Pustejovsky & Tipton, 2021).

To estimate the overall impact of pollination treatments on yield stability, we used models with only random effects (a null model, analogous to an LMM with no fixed effects; these are models CVR0 and VR0 in Table 2) and the arm‐based model described above (SD0). We then added moderators (fixed effects) to the null model to quantify how different factors change the stability ratio between animal‐pollinated and auto‐pollinated plants. These moderators were the crop species (CVR1 and SD1), the spatial scale of aggregation (CVR2 and SD2), pollination treatment type (CVR5 and CVR6) and pollinator activity level (CVR7; see sections below and supplementary material).

**TABLE 2:** Summary of models in order of appearance in manuscript. See supplementary information for matching R code for each model. The number of levels for each fixed or random effect is provided in brackets

Model	Dependent variable	Moderators (Fixed effects)	Random effects
CVR0	lnCVR	–	Publication (47), experimental comparison (215), effect size (498)
SD0	lnSD	ln(mean) (498)	
RR0	lnRR	–	
VR0	lnVR	–	
CVR1	lnCVR	Crop species (3; apple, oilseed rape, faba bean)	
SD1	lnSD	ln(mean) + crop species (3)	
CVR2	lnCVR	Scale (4; flower, plant, plot, site)	
SD2	lnSD	ln(mean) + scale (4)	
CVR3	lnCVR	lnRR (498)	
CVR4	lnCVR	z‐score standardised mean yield of animal‐pollinated plants (497)	Publication (46), experimental comparison (214), effect size (497)
RR1	lnRR	z‐score standardised mean yield of auto‐pollinated control plants (498)	Publication (47), experimental comparison (215), effect size (498)
CVR5	lnCVR	Pollinator type (6; bumblebee, hand, honeybee, hoverfly, solitary, open‐pollinated)	
CVR6	lnCVR	Pollinator type (2; other, open‐pollinated)	
CVR7	lnCVR	Pollination intensity (2; high, low)	Publication (11), experimental comparison (46), effect size (104)

We fitted all the meta‐analytic models with maximum likelihood, ML, for model comparisons and re‐ran with restricted maximum likelihood, REML, to produce the model outputs that we report in the manuscript. We used a multilevel version of index, *I* ^2^ (see Nakagawa & Santos, 2012) to quantify the total level of heterogeneity in our effect size ratios and heterogeneity associated with different levels of clustering (the random effects, see below) (Higgins & Thompson, 2002). We used marginal *R* ^2^ to quantify how much heterogeneity is explained by different moderators (the fixed effects; Nakagawa & Schielzeth, 2013). A summary of each model is presented in Table 2, while more details and complete code for the analysis are presented in the supplementary material.

### Spatial scale

Often in meta‐analyses, the sample size *n* is identical between experimental units and the number of individual animals or plants. The means and variances reported in the publications that we analysed were calculated at different spatial scales; some authors calculated means and variances between individual plants (e.g. St‐Martin & Bommarco, 2016) while others calculated means and variances using a single value per field site (e.g. Mallinger & Gratton, 2015; Table 1). The reported variance that we use in our stability ratios can therefore represent yield stability between flowers on a plant, between plants, between plot or cohort means (which we consider equivalent), or between field site means. Impacts of pollination on yield stability at these different spatial scales have different implications for agricultural producers and probably have different mechanisms. We tested the scale at which variance was calculated as a moderator (CVR2 and SD2).

When data provided in publications were subject to nonindependence that could not be accounted for using our random effects structure as described above, we used raw data from the publications if it was available, and aggregated it at the largest scale to avoid nonindependence (this applies to 26 of 498 effect sizes).

### Resource limitation

We tested whether resource limitation (Garibaldi et al., 2018) may be driving changes in yield stability: We hypothesised that when crop yield is increased by animal pollination, yield becomes increasingly limited by other factors, causing clustering around the maximum potential yield in that context, which results in increased stability (a ceiling effect, see O'Dea et al., 2018). These limiting factors could include agronomic constraints such as nutrient availability, and/or physiological constraints, such as the number of ovules per flower. To test this we used additional models to quantify the relationship between the yield stability ratio (lnCVR) and the yield benefit of animal pollination (lnRR; CVR3) or the standardised yield of animal‐pollinated plants (CVR4). Resource limitation would be indicated if (i) lnCVR was negatively associated with lnRR; when animal pollination has a relatively greater yield benefit (and animal‐pollinated plants are more likely to be close to the maximum yield) the CV of animal‐pollinated plants is relatively smaller than control plants, and (ii) lnCVR was negatively associated with yield production in animal‐pollinated plants; when animal‐pollinated plants are closer to the maximum potential yield, the CV between them is relatively smaller. Further, to test whether the benefit of pollination to yield is greatest in contexts where auto‐pollinated plants are less productive (and further from the maximum potential yield) we compared the level of pollination benefit (lnRR) and the standardised yield of auto‐pollinated plants (RR1). Crop yield varies between contexts (Garibaldi et al., 2018), so to enable comparisons across different effect sizes we standardised yield using z‐scores within each combination of crop, response measure (how yield was measured) and spatial scale. For simplicity, we ran these additional models as univariate models, and therefore assume that there is no sampling error in the explanatory variables.

### Pollinator activity

Several publications in our analysis were conducted to measure differences in pollination service at different locations by comparing the yield of open‐pollinated plants with control plants enclosed in cages at each location (e.g. Perrot et al., 2018; Toivonen et al., 2019; Zou et al., 2017). Based on previous research (Garibaldi, Steffan‐Dewenter, et al., 2011; Lázaro & Alomar, 2019), we hypothesised that differences (instability) in pollination service between locations would make yield less stable in these open pollination experiments, in comparison to experiments where pollinator activity was maximised by introduction of pollinating insects or hand‐pollination inside cages (e.g. Bishop et al., 2016). We tested animal pollination treatment type as a moderator (CVR5) and compared open‐pollination to other pollination treatments (CVR6). We also tested the effect of pollination activity level (CVR7), which was experimentally manipulated in 11 of the publications (e.g. there was more than one level of pollination activity in these studies aside from the auto‐pollinated control treatment) in two different ways; we assumed that animal pollination plus hand‐pollination (used to test for pollination deficits) represented greater pollinator activity than animal pollination only, and we assumed that a greater density of pollinating insects resulted in greater pollination activity.

### Sensitivity analyses

For the stability analyses, it was not possible to use common tests for publication bias (see Nakagawa et al., 2021) because these tests use the sampling error, upon which our effect sizes are based. We also consider it unlikely that variance is subject to the same publication biases as mean effects. We were able to test for time lag bias (Koricheva & Kulinskaya, 2019) by including publication year as a moderator in the lnCVR analysis, we found there was no significant change in lnCVR over time (supplementary material). To determine whether our lnCVR findings were robust to the exclusion of individual publications, we used leave‐one‐out analyses where we ran our random effect only (null) model multiple times leaving out one publication at a time and observed how this affected the estimate; leaving individual publications out had little impact on our findings (Figure S4). We also tested the influence of including more than one effect size from each experimental comparison using another leave‐one‐out analysis, where we ran our null model multiple times randomly selecting a different effect size from each experimental comparison; this had little impact on our findings (supplementary material). We used Egger's regression with effective sample size (Nakagawa et al., 2021) to test for publication bias in the log response ratio (lnRR) analysis (which compares mean effects) and found no significant evidence of publication bias (supplementary material).

## RESULTS

### Overall effects

Overall, animal pollination increased yield stability across space by 32% (95% confidence interval 22 to 40%; model CVR0 in Table 2) with the coefficient of variation in yield lower among groups of animal‐pollinated plants compared to auto‐pollinated control plants. There was low heterogeneity in this yield stabilising effect of pollination across the experimental comparisons we synthesised (*I* ^2^_total_ 39%) and a small amount of unexplained heterogeneity (*I* ^2^_residual_ 11%). In comparison, our lnSD analysis estimated that yield of animal‐pollinated plants is 23.3% (15–31%; SD0) more stable than controls. We also tested how animal pollination treatments affected mean crop yield (using the log ratio of means, lnRR) and absolute stability (using the log ratio of variances, lnVR, which is not mean‐corrected). On average, animal pollination increased mean crop yield by 104% (66 to 152%; RR0) and this was associated with a decrease in absolute yield stability (an increase in variance) of 29% (14–41%; VR0). Other results relating to absolute stability are reported in the supplementary material.

### Crop species

**FIGURE 1:**
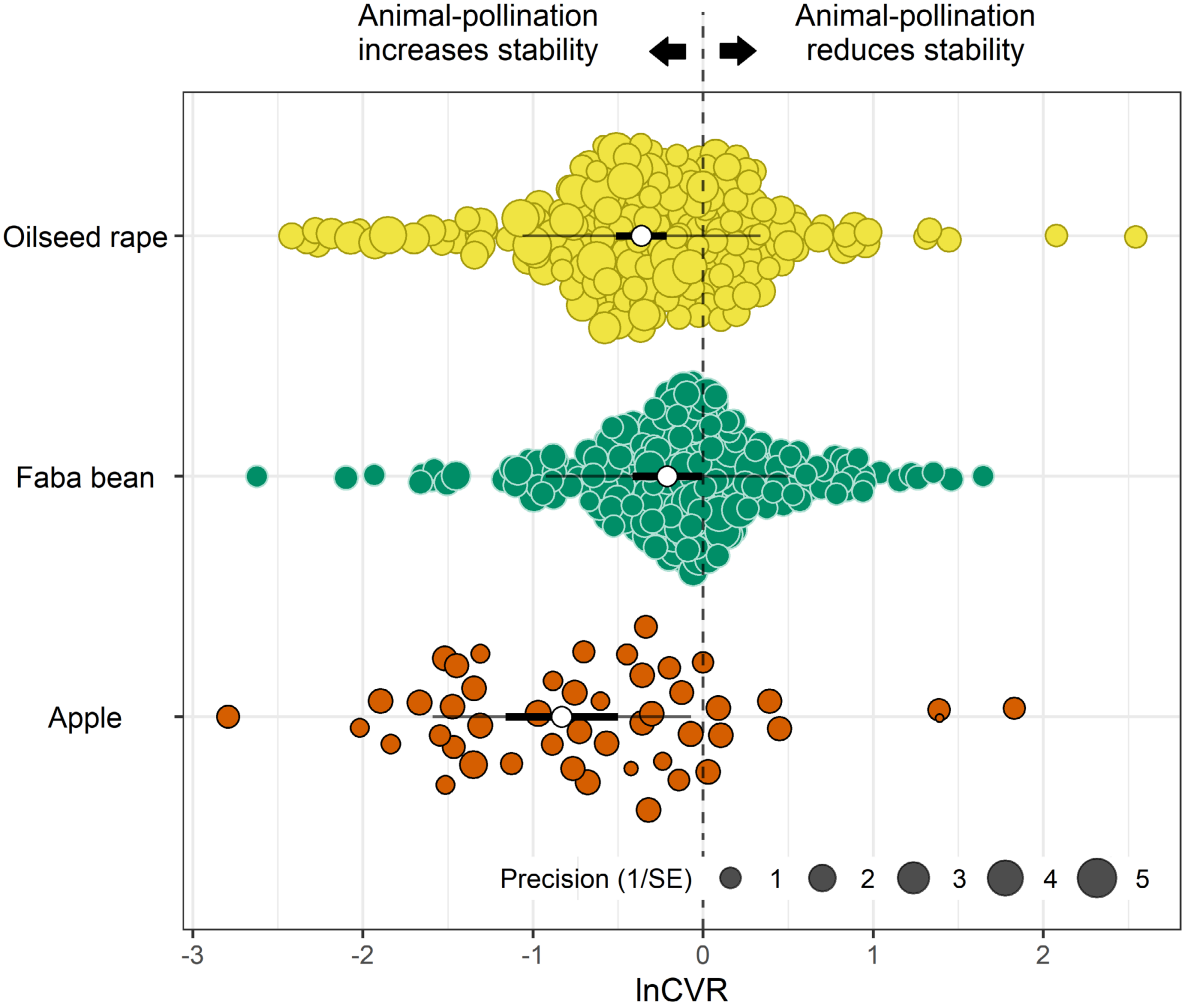
Effect of crop species on the yield stability ratio (lnCVR) between animal‐pollinated and auto‐pollinated control plants. The white open points show the mean effect size for each crop species, the bold lines show the 95% confidence intervals and the thin lines show the 95% prediction intervals.

The effect of animal pollination on spatial yield stability differed between crop species (Figure 1; likelihood ratio test comparing model with crop as moderator (CVR1) versus null model (CVR0) *p* = 0.009) with the largest increase in apple (56%), followed by oilseed rape (30%) and the smallest in faba bean (19%; CVR1). Corresponding lnSD estimates for each crop were more similar (apple = 29.3%, oilseed rape = 27.7% and faba bean = 14.2%; SD1) indicating that the particularly large lnCVR estimate for apple is partially due to over‐correction of the mean–variance relationship.

### Spatial scale

**FIGURE 2:**
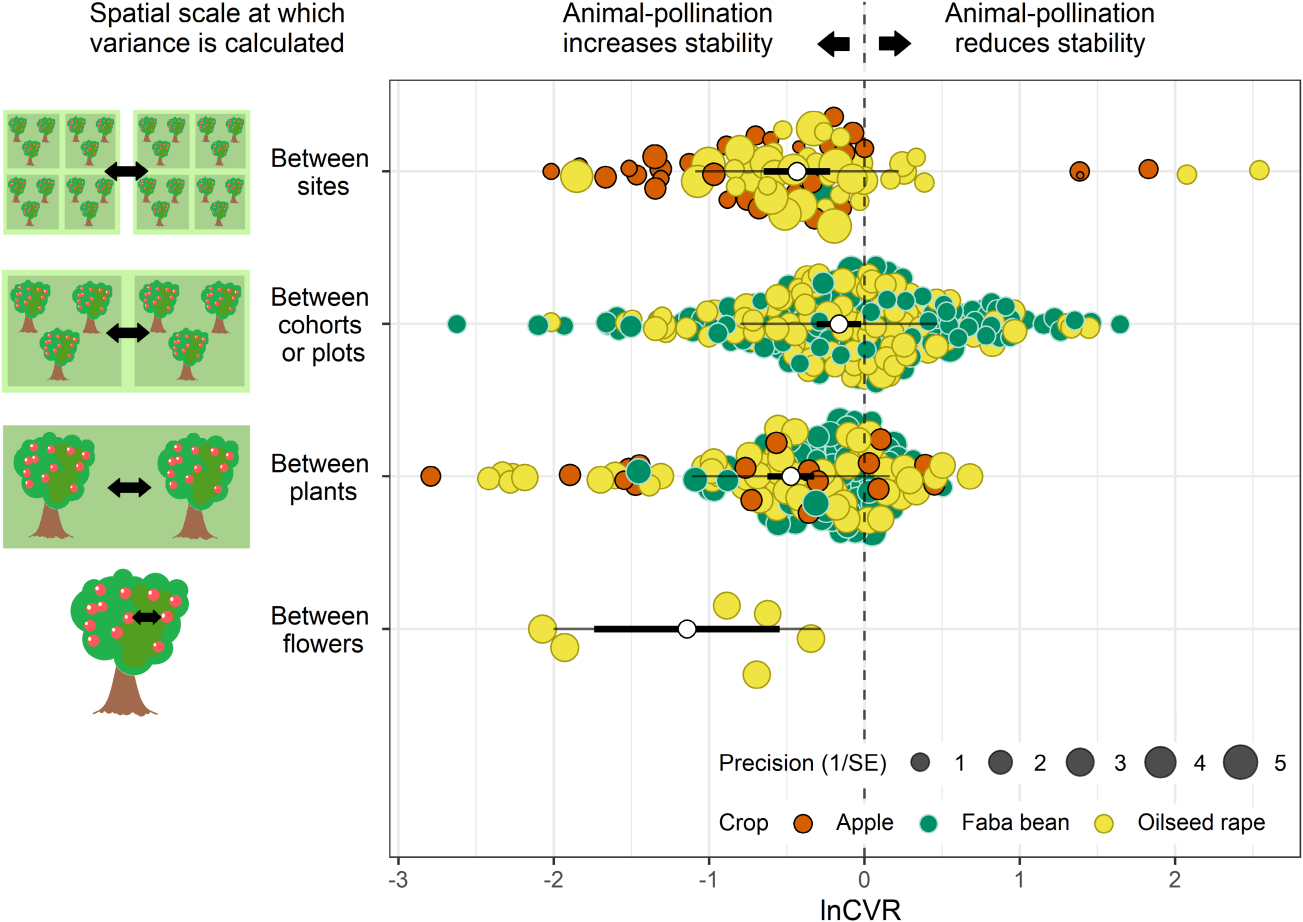
Effect of spatial scale at which mean and variance have been calculated on the yield stability ratio (lnCVR) between animal‐pollinated and auto‐pollinated control plants. The white open points show the mean effect size for each spatial scale, the bold lines show the 95% confidence intervals the thin lines show the 95% prediction intervals.

Our analyses show that biotic pollination increased spatial stability of crop yield at all tested scales (Figure 2) though the size of this effect differed between scales (LRT CVR2 vs. CVR0; *p* < 0.001). The greatest increase in stability with animal pollination was between‐flowers, and the lowest was between‐plots within fields. Notably, there was a 35% increase in stability with animal pollination at the between‐site scale (*p* < 0.001; CVR2). A lnSD analysis at the site scale produced the same 35% estimate (*p* < 0.001; SD2).

### Resource limitation

**FIGURE 3:**
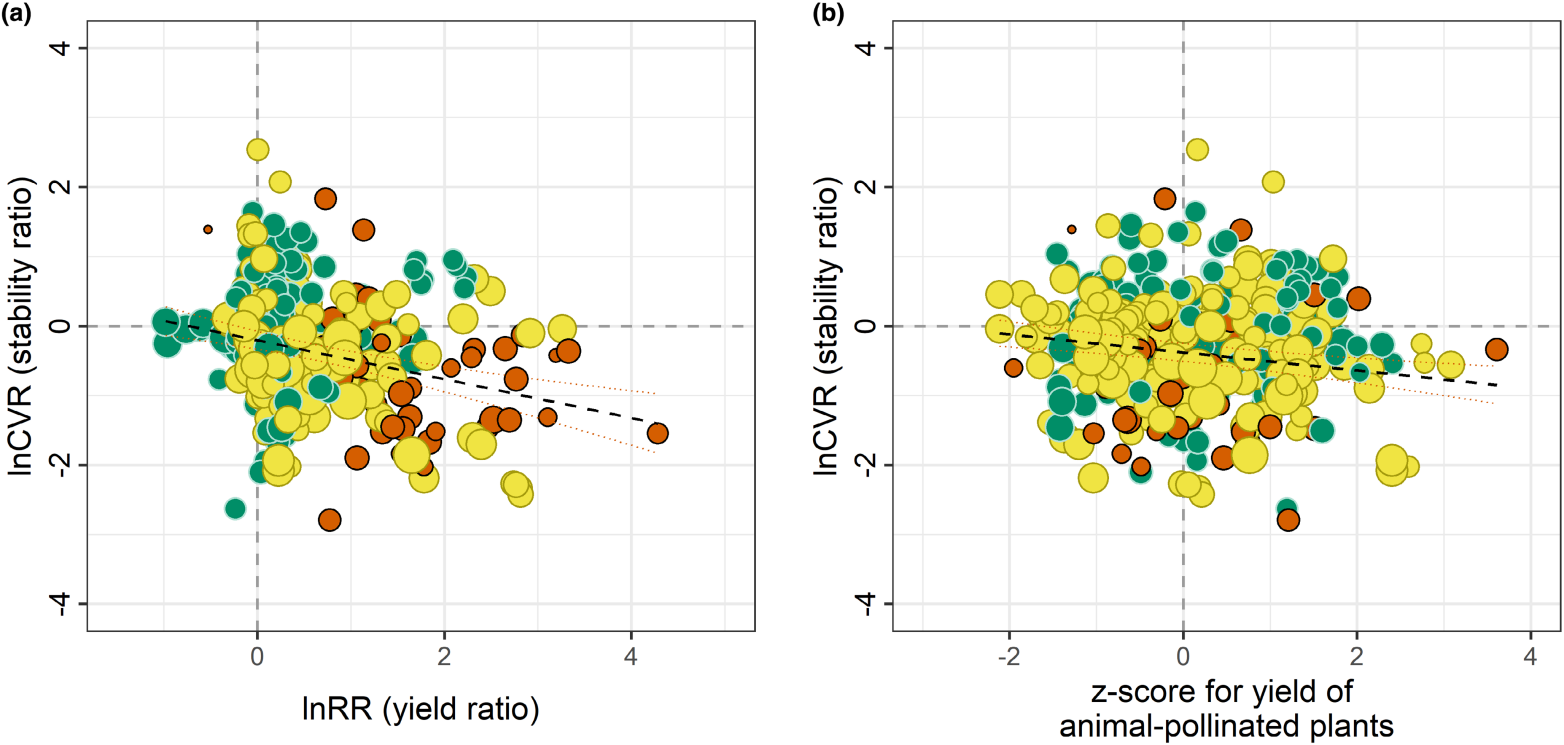
Relationships between the yield benefit of animal pollination (lnRR) or the z‐transformed yield of animal‐pollinated plants and the yield stability ratio (lnCVR). Negative lnCVR indicates stability increase with animal pollination. Positive lnRR indicates yield increase with animal pollination. Lines are fits from univariate regression models and 95% confidence intervals. (a) Relative stability ratio vs. yield benefit; (b) relative stability ratio versus z‐transformed yield of animal‐pollinated plants, standardised within each combination of response metric, crop and spatial scale.

In support of our resource limitation hypothesis, we found a significant negative relationship between the yield benefit of animal pollination and the yield stability ratio (slope −0.28, *p* < 0.001; CVR3); meaning that when the yield benefit of pollination is greater (lnRR more positive), the yield of animal‐pollinated plants is relatively more stable than control plants (lnCVR more negative; Figure 3a). We also found a significant negative relationship between the standardised yield of plants receiving animal pollination and the yield stability ratio; when animal‐pollinated plant yield was relatively high, animal‐pollinated plants were more stable (lnCVR more negative; slope −0.13, *p* < 0.001, Figure 3b; CVR4). Finally, we found a significant negative relationship between the standardised yield of auto‐pollinated control plants and the yield benefit of animal pollination (lnRR), the yield benefit of pollination is greater when the yield is relatively lower in auto‐pollinated control plants (slope −0.15, *p* < 0.001, Figure S3; RR1).

### Pollinator activity

All animal pollination treatments resulted in a similar increase in yield stability (LRT CVR5 vs. CVR0, *p* = 0.46; LRT CVR6 vs. CVR5, *p* = 0.9). We found no significant effect of pollinator activity on the stability ratio (*p* = 0.7; CVR7) between animal‐pollinated plants and auto‐pollinated controls.

## DISCUSSION

Our results provide clear evidence that animal pollination consistently increases crop yield stability across a range of spatial scales in three globally important and representative crop species. Many studies have demonstrated that animal pollination is beneficial to crop yield production (Aizen et al., 2009; Breeze et al., 2016; Klein et al., 2007; Potts et al., 2016). We have used an approach akin to these pollination dependence assessments (see Breeze et al., 2016) to show that the yield of plants receiving animal pollination is on average 32% more stable across space than control plants relying on wind pollination or autogamy. Stable and predictable production of nutritious food is a necessity for global food security and for food producers. Animal‐pollinated crops constitute 75% of the world's major crops (Klein et al., 2007) and are of global importance to human nutrition (Smith et al., 2015), yet crops are grown in different conditions and receive different agricultural management across the world, making observational comparisons of their yield stability difficult (Garibaldi, Aizen, et al., 2011; Oguro et al., 2019). Using recent methodological developments in meta‐analysis we have synthesised 215 controlled experimental comparisons across 20 countries to illustrate this additional benefit of pollination services.

Our findings indicate that managing pollination services to achieve high crop yield will also result in more stable crop yield. In our study crops, greater yield benefits of animal pollination were associated with greater yield stability. Our results indicate that animal pollination increases spatial yield stability due to a ceiling effect (O'Dea et al., 2018) which results from resource limitation. According to the multiple limitation hypothesis (Garibaldi et al., 2018), crop yield is concurrently limited by different factors, though the relative extent of limitation due to a single factor may relate to its demand. As animal pollination increases the number of fertilised ovules (sinks) in plants, the ability of plants to mature these additional seeds and fruits becomes increasingly constrained by other factors resulting in a clustering of yield at the maximum yield potential, resulting in greater similarity between plants and greater stability. In support of this, we found that the stability ratio between animal‐pollinated and auto‐pollinated plants is larger in experimental comparisons where the standardised yield of animal‐pollinated plants is relatively high (and more likely to be constrained by other factors such as nutrients; Garratt, Bishop, et al., 2018). Second, we found that the yield benefit of animal pollination is greatest in experimental comparisons where the standardised yield of auto‐pollinated control plants is relatively low (and less likely to become constrained by other factors).

Experimental evidence suggests that animal pollination most often interacts positively (synergistically) with other yield‐enhancing factors (Tamburini et al., 2019) and that plants with more resources (e.g. greater soil nutrient availability; Garratt, Bishop, et al., 2018) are better able to support additional seeds and fruits resulting from animal pollination. Contrary to our findings, this would imply that enhancing animal pollination services could exaggerate existing yield differences between locations and therefore reduce yield stability across space. However, synergistic interactions have typically been identified in experimental conditions where resource availability has been manipulated (Tamburini et al., 2019). In reality, crops are frequently resource limited (Mueller et al., 2012) and the experimental comparisons included in our analysis came from diverse countries and contexts and often tested the effect of animal pollination in a conventionally managed, commercial context. An implication of our results is that the role of animal pollination in yield stability is particularly important in developing countries, where crops are more frequently resource limited, and where yield stability is more directly linked to food security via subsistence agriculture (Garibaldi et al., 2018; Mueller et al., 2012).

Stability of pollinator activity has previously been linked to stability of yield (Garibaldi, Steffan‐Dewenter, et al., 2011; Lázaro & Alomar, 2019) but we found no difference in stability between experimental comparisons with potentially variable levels of animal pollination (e.g. open‐pollination treatments) and those that maximised pollination (e.g. pollinating insects released in a cage or hand pollination). Several studies also experimentally applied different levels of animal pollination by performing additional pollination treatments (Garratt, Evans, et al., 2014) or manipulating pollinator density (Garratt, Coston, et al., 2014), but we found no difference in the stability ratio between low pollinator activity vs. control and high pollinator activity vs. control comparisons. Auto‐pollinated controls in the experimental comparisons that we synthesised represent a worst‐case scenario where all animal pollinators have been lost. Across different experimental animal pollination treatments, pollinator activity appears to have been above a minimum‐adequate level to differentiate animal‐pollinated and control plants (Hünicken et al., 2021). Similarly, increased honeybee activity has recently been shown to increase mean yield, but there was a nonlinear relationship where yield did not increase beyond an optimal level of pollinator activity (Rollin & Garibaldi, 2019). This again may be due to a ceiling effect, with yield benefits of enhanced animal pollination being limited by other resource constraints. Pollinator management needs to be considered alongside other agronomic practices with an understanding of which combination of factors are limiting yield in each context (Garratt, Bishop, et al., 2018).

Temporal (between‐year) yield stability is arguably more important to individual farmers than spatial stability. Only four publications that we identified presented data from three or more years, making it impossible to test impacts of animal pollination on temporal yield stability. We did, however find that animal pollination treatments made yield more stable between fields in different locations, despite likely differences in environmental conditions (e.g. weather; Bishop et al., 2016), management practices, or resource availability. If we take a space for time substitution approach and assume that variance between sites is representative of variance between years (Johnson & Miyanishi, 2008; Walker et al., 2010) our results indicate that animal pollination increases the temporal stability or the predictability of yield. Such a benefit of animal pollination is valuable to food producers who strive for consistent yield from year to year and from field to field (Barah et al., 1981; Eskridge, 1990).

The monetary value of animal pollination services ($235–577 billion globally each year in 2016; Potts et al., 2016) has been used to promote agronomic management for animal pollinators and to prioritise their conservation (Dicks et al., 2016). The additional benefits that animal pollination provides for crop yield stability that we demonstrate here have not yet been accounted for in economic or natural capital assessments (Breeze et al., 2016). Quantifying stability benefits in such terms would provide further justification for targeted and long‐term investment in measures to protect pollinators.

### AUTHORSHIP STATEMENT

JB conceived the study, conducted the literature review and data extractions and wrote the manuscript. JB and SN undertook the analyses and produced the figures. All authors contributed to the interpretation of the results and editing the manuscript.

### PEER REVIEW

The peer review history for this article is available at https://publons.com/publon/10.1111/ele.14069.

## Supporting information


Appendix S1

## Data Availability

This is a meta‐analysis of data from published articles. We have archived the data supporting the results (that we extracted from the published articles) using Open Science Framework (DOI: 10.17605/OSF.IO/T5EKB) and our code is available in the supporting information.
