# Supplementary material for: Animal pollination increases stability of crop yield across spatial scales
Source: Ecol Lett. 2022 Jul 17;25(9):2034–47. doi: 10.1111/ele.14069 (PMC9544623; doi:10.1111/ele.14069)
Supplement: Supplementary file 1 — Appendix S1 [file ELE-25-2034-s001.zip › ELE_14069_supplementary info_r2.pdf]

Supplementary Information - Animal pollination increases stability of crop yield across spatial scales

Jacob Bishop, Michael P.D. Garratt & Shinichi Nakagawa

June 2022

Contents

Introduction 2

Identifying literature 2

Preparation 4

Calculating effect sizes 5

Overview of the effect size measures . . . 6

Calculate lnCVR . . . 6

Calculate lnVR . . . 6

Calculate lnRR . . . 7

Setup for lnSD analysis . . . 7

Random effects models to find overall effects of animal-pollination 8

CVR0 - Random effects models lnCVR - relative stability . . . 8

VR0 - Random effects models lnVR - absolute stability . . . 10

RR0 - Random effects models lnRR - pollination benefit . . . 12

SD0 - lnSD (arm-based) analyses . . . 13

Multilevel meta-analysis models with moderators 14

lnCVR models - effect of moderators on relative stability . . . 14

CVR2 - lnCVR - scale . . . 14

CVR1 - lnCVR - crops . . . 16

CVR5 & CVR6 - lnCVR - pollination treatment type . . . 17

CVR7 - lnCVR - pollinator activity . . . 20

lnCVR - how yield is measured . . . 22

lnVR models - effect of moderators on absolute (not mean-adjusted) stability . . . 24

lnVR - scale 24
lnVR - crops 25
lnVR - pollination treatment type and pollination effort 26
lnVR - how yield is measured 28
lnRR - effect of moderators on pollination dependence 29
lnRR - how yield is measured 29
lnSD models - effect of moderators on stability 30
SD1 - lnSD - crops 30
SD2 - lnSD - scale 32
Relationships between estimators and testing for ceiling effects **33**
Relationships between pollination benefit and yield stability 34
CVR3 - Relative stability and pollination benefit; lnCVR vs lnRR 34
Absolute stability and pollination benefit; lnVR vs lnRR 35
CVR4 & RR1 - Stability and mean yield 36
Sensitivity analyses **39**
Leave-one-out sensitivity analysis for lnCVR 39
Egger regression for lnRR 40
Time lag bias test for lnCVR 43
R Session Information **45**

Introduction

This electronic supplementary material provides a step by step description and explanation of our methodology, alongside information about the publication bias and sensitivity analyses that we conducted. Please also see provided spreadsheets which include our dataset, systematic review steps, and a PRISMA-EcoEvo checklist. See the main manuscript for relevant references.

Identifying literature

One person (JB) conducted the literature search and made all decisions regarding which studies to include or exclude. As described in the main text, We used four recent systematic reviews on biotic pollination and crop yield as the starting point for our literature search. We updated each of these systematic reviews up to October 2020 by searching Web of Science with the same search strings as each original review. JB extracted data from the publications as described in the manuscript, 10% of the extracted data were checked by MG.

Table 1: Summary of systematic reviews that we updated, combined and used in the present analysis. The count of publications selected by the systematic reviews is larger than the count of articles that we used in our analysis, as not all publications met our inclusion criteria.

Systematic review	Search terms	Search databases	Publication we used count
Bishop & Nakagawa 2021	"faba" AND "pollination"	Scopus, Web of Science, Google Scholar	14
Ouvrard & Jacquemart 2019	"Brassica napus" AND "pollination" AND "yield"	Scopus, Web of Science, Google Scholar	16
Woodcock et al 2019	"Oilseed rape" OR "Canola" OR "Rapeseed" OR "Brassica napus" AND "Pollination/Pollinator(s)" AND "Yield"	Web of Science	+2
Pardo & Borges 2020	"insect pollination" AND "apple orchard"; "pollination" AND "apple orchard"; "insect pollination" AND "apple orchard" AND "management"; "insect pollination" AND "apple orchard" AND "landscape"; "apple" AND "pollinators"; "apple" AND "honeybees"; "apple" AND "bumblebees"; "apple" AND "wild bees"; "apple" AND "hoverflies" "apple" AND "pollination services"	Scopus, Web of Science, Google Scholar	10

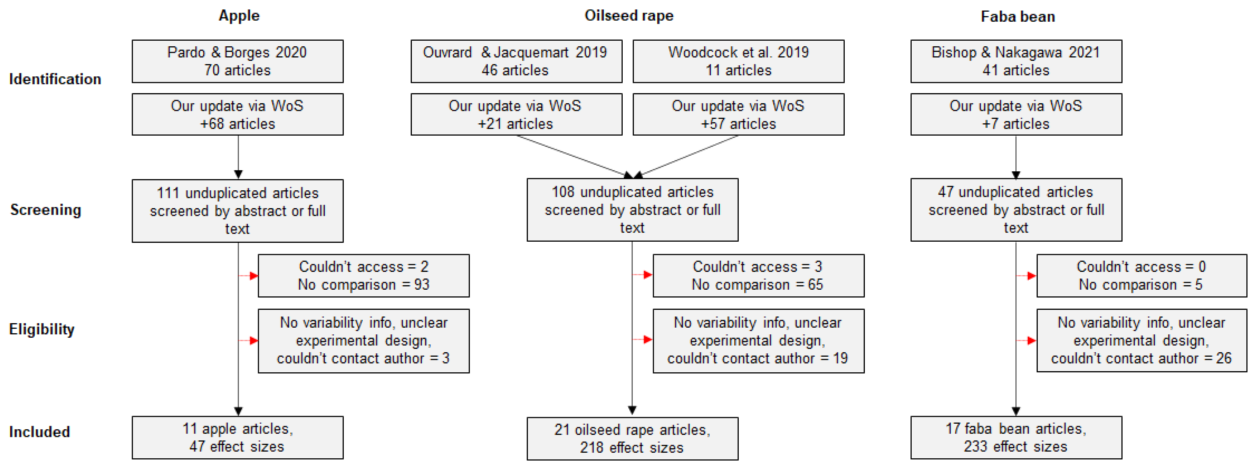

Figure S1: PRISMA (Preferred Reporting Items for Systematic Reviews and Meta-Analyses) diagram.

Preparation

First we install and/or load necessary packages

```
library(metaAidR)
library(orchaRd)
library(metafor)
library(devtools)
library(tidyverse)
library(patchwork)
library(R.rsp)
library(tibble)
library(cowplot)
library(metafor)
library(corpcor)
library(here)
library(MCMCglmm)
```

Then we load some additional functions that we prepared

```
source(here("R/functions.R"), chdir = TRUE)
```

Load the data

```
data1 <- read.csv(here("data", "combined_dataset_v2.csv"),
    skip=1, na.strings=c("","NA"), stringsAsFactors=TRUE)
```

Clean the data; what we are doing here is removing any effect sizes where n is less than 1 or where SD is zero. We also remove effect sizes from our literature search that we judged not to be relevant; for example we do not look at yield quality data (seed or fruit size) in this work.

```
# remove effect sizes where n less that 1 or SD is zero or NA, we lose 5 values
data2 <- droplevels(subset(data1,npoll>1))
data2 <- droplevels(subset(data2,sdpoll!=0))
data2 <- droplevels(subset(data2,sdpoll!="NA"))
data2 <- droplevels(subset(data2,sdexc!=0))
data2 <- droplevels(subset(data2,sdexc!="NA"))

# remove response types with unclear relation to yield production
levels(data2$response)
data2 <- droplevels(subset(data2,response!="quality"))
data2 <- droplevels(subset(data2,response!="podlength"))
data2 <- droplevels(subset(data2,response!="width"))
data2 <- droplevels(subset(data2,response!="tgw"))
```

Now some counting of different things for reporting in the manuscript.

How many publications do we have in our final dataset?

```
length(levels(factor(data1$study_id)))
```

```
## [1] 47
```

How many individual experimental comparisons do we have in our final dataset? (We find this by counting the number of multiple outcome clusters - each experimental comparison is given a unique identifier - as we explain in the manuscript, experimental comparisons can contribute multiple data rows because different response variables (e.g. yield, seed number; multiple endpoints) are measured).

```
length(levels(factor(data2$multiple_endpoint_clusterID)))
```

```
## [1] 215
```

How many countries do our effect sizes come from and which countries are represented?

```
length(levels(factor(data2$country)))
```

```
## [1] 20
```

```
levels(factor(data2$country))
```

```
##  [1] "algeria"     "argentina"   "australia"   "belgium"     "brazil"
##  [6] "canada"      "chile"       "china"       "finland"     "france"
## [11] "germany"     "italy"       "netherlands" "new zealand" "pakistan"
## [16] "poland"      "sweden"      "switzerland" "uk"          "usa"
```

Calculating effect sizes

First, we see to what extent ln(mean) and ln(sd) are correlated. We see that they are strongly associated (fig. S2), indicating that some correction of the mean-variance relationship is required (see below).

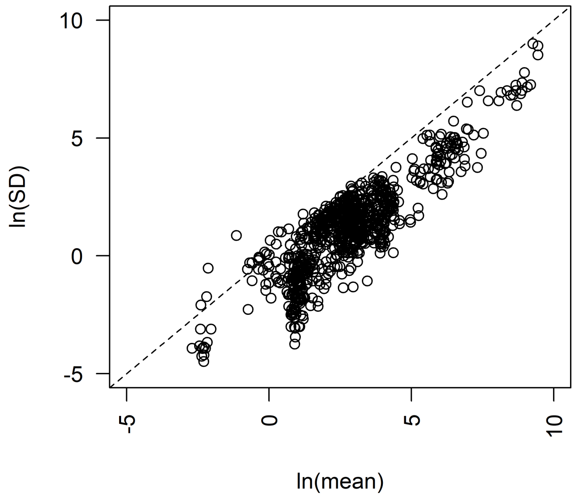

Figure S2: ln(mean) and ln(sd)

Overview of the effect size measures

For our analyses we use lnCVR (log transformed ratio of the coefficient of variation). lnCVR is useful because pollination is known to affect the mean (and potentially the variance, as we are testing in this study). We also use lnVR (log transformed ratio of the standard deviations), lnRR (log transformed ratio of the means), and perform lnSD models (where changes in ln(SD) are explicitly modeled in terms of changes in ln(Mean)). See the main manuscript for a more detailed explanation of these effect sizes and why we use them. We use new estimators of these effect sizes that can account for differences in sample size between the two groups. While calculating our effect size measures we also calculate variance co-variance matrices to account for dependence that results from treatment effects being compared to a shared control (e.g. where two different animal-pollination treatments are performed, and compared to the same auto-pollinated control group).

Calculate lnCVR

```
data2$lncvr.yi <- with(data2,lnCVR2(CMean=meanexc,
                          CSD=sdexc,
                          CN=nexc,
                          EMean=meanpoll,
                          ESD=sdpoll,
                          EN=npoll))[,1]
data2$lncvr.vi <- with(data2,lnCVR2(CMean=meanexc,
                                     CSD=sdexc,
                                     CN=nexc,
                                     EMean=meanpoll,
                                     ESD=sdpoll,
                                     EN=npoll))[,2]

# adding control variance for shared control VCV
data2$cV.lnCVR <- with(data2,cV.lnCVR(CMean=meanexc,
                                      CSD=sdexc,
                                      CN=nexc))

# note: rows that share a common "sharedcontrol_clusterID" share a common control
data2 <- data2[order(data2$sharedcontrol_clusterID),]

# shared control = true
V.d2.shared.lnCVR <- VCV.shared(data=data2, cV="cV.lnCVR",
    V = "lncvr.vi", cluster="sharedcontrol_clusterID", obs="info_id")
is.positive.definite(V.d2.shared.lnCVR) #TRUE
```

Calculate lnVR

```
data2$lnvr.yi <- with(data2,lnVR2(CMean=meanexc,
                                   CSD=sdexc,
                                   CN=nexc,
                                   EMean=meanpoll,
                                   ESD=sdpoll,
                                   EN=npoll))[,1]
data2$lnvr.vi <- with(data2,lnVR2(CMean=meanexc,
                                   CSD=sdexc,
```

```
                                        CN=nexc,
                                        EMean=meanpoll,
                                        ESD=sdpoll,
                                        EN=npoll))[,2]

# need to make VCV for shared conrol for lnVR
data2$cV.lnVR <- with(data2, cV.lnVR(CN = nexc))
V.d2.shared.lnVR <- VCV.shared(data=data2, cV="cV.lnVR",
    V = "lnvr.vi", cluster="sharedcontrol_clusterID", obs="info_id")
is.positive.definite(V.d2.shared.lnVR)
```

Calculate lnRR

Note that this is setup so a positive yi means improvement with insect pollination.

```
data2$lnrr.yi <- with(data2,lnRR2(CMean=meanexc,
                                  CSD=sdexc,
                                  CN=nexc,
                                  EMean=meanpoll,
                                  ESD=sdpoll,
                                  EN=npoll))[,1]
data2$lnrr.vi <- with(data2,lnRR2(CMean=meanexc,
                                  CSD=sdexc,
                                  CN=nexc,
                                  EMean=meanpoll,
                                  ESD=sdpoll,
                                  EN=npoll))[,2]

# for VCV shared control
data2$cV.lnRR <- with(data2,cV.lnRR(CMean=meanexc,
                                    CSD=sdexc,
                                    CN=nexc))
# shared control = true
V.d2.shared.lnRR <- VCV.shared(data=data2, cV="cV.lnRR",
     V = "lnrr.vi", cluster="sharedcontrol_clusterID", obs="info_id")
is.positive.definite(V.d2.shared.lnRR) #TRUE
```

Setup for lnSD analysis

Creating a long-format data for lnSD analysis

```
data_temp1 <- with(data2, lnSD_lnM(Mean = meanpoll,
                                   SD = sdpoll,
                                   N = npoll))
data_temp2 <- with(data2, lnSD_lnM(Mean = meanexc,
                                   SD = sdexc,
                                   N = nexc))
# combining the two data frames
data_long <- rbind(cbind(data2, data_temp1), cbind(data2, data_temp2))

# adding two new columns to indicate pollinated or excluded
```

```
data_long$obs <- factor(1:dim(data_long)[[1]])
data_long$treatment <- as.factor(rep(c("pollinated", "excluded"),
                                     each = dim(data_long)[[1]]/2))

dim(data_long)
```

Random effects models to find overall effects of animal-pollination

CVR0 - Random effects models lnCVR - relative stability

Run first model, no moderators, random effects for study, multiple outcome cluster, and residual

```
nullmod <- rma.mv(yi=lncvr.yi,
    V=V.d2.shared.lnCVR,
    random=list(~1|study_id,~1|multiple_endpoint_clusterID,~1|info_id),
    data=data2,method="ML", sparse = TRUE)
summary(nullmod)
AIC(nullmod) # 920.115
```

As suggested by the referees, we try adding in cultivar as a random effect. We find that the addition of cultivar does not improve the model performance so we do not include it in the models.

```
# get out combinations of cultivar
study.cult <- unique(data2[,c('study_id','cultivar')])

nullmod.cult <- rma.mv(yi=lncvr.yi,
    V=V.d2.shared.lnCVR,
    random=list(~1|study_id,~1|cultivar,~1|multiple_endpoint_clusterID,~1|info_id),
    data=data2,method="ML", sparse = TRUE)
summary(nullmod.cult)
AIC(nullmod.cult) # 922.077
anova(nullmod.cult,nullmod) # no difference between models
```

Re-run the null model with REML for reporting in manuscript

```
nullmod.reml <- rma.mv(yi=lncvr.yi,
    V=V.d2.shared.lnCVR,
    random=list(~1|study_id,~1|multiple_endpoint_clusterID,~1|info_id),
    data=data2,method="REML", sparse = TRUE)
summary(nullmod.reml)
```

```
##
## Multivariate Meta-Analysis Model (k = 498; method: REML)
##
##    logLik   Deviance        AIC        BIC       AICc
## -454.7830   909.5661   917.5661   934.4004   917.6474
##
## Variance Components:
##
##             estim    sqrt  nlvls  fixed                       factor
```

```
## sigma^2.1  0.0926  0.3044     47     no                     study_id
## sigma^2.2  0.0102  0.1010    215     no  multiple_endpoint_clusterID
## sigma^2.3  0.0424  0.2059    498     no                      info_id
##
## Test for Heterogeneity:
## Q(df = 497) = 772.7755, p-val < .0001
##
## Model Results:
##
## estimate      se     zval    pval    ci.lb    ci.ub
##  -0.3786  0.0638  -5.9301  <.0001  -0.5037  -0.2534  ***
##
## ---
## Signif. codes:  0 '***' 0.001 '**' 0.01 '*' 0.05 '.' 0.1 ' ' 1
```

Get estimates from the REML model for reporting in the manuscript. We can convert our ratio measure to the *percentage change in stability* by using 1-exp(lnCVR). Our overall estimate is that pollinated plants are c.32% less variable (or c.32% more stable) than excluded plants.

```
1 - exp(nullmod.reml$b[[1]])
```

```
## [1] 0.3151606
```

Confidence intervals

```
## [1] 0.3957046
## [1] 0.2238814
```

Get I2, our heterogeneity measure for the null model

```
##                        I2_total                     I2_study_id
##                      0.39343182                      0.25095436
## I2_multiple_endpoint_clusterID                      I2_info_id
##                      0.02760473                      0.11487273
```

As suggested by the referees, we add a sensitivity analysis where we run a model choosing one effect size from each experimental comparison that was conducted. As described in the main manuscript, there were 47 publications, 215 experimental comparisons in total, and 498 effect sizes. There were more effect sizes than experimental comparisons because publications often reported yield outcomes across several different response measures (e.g. 'yield mass', 'seed number'). We account for dependence between these effect sizes with our random effects structure (we include 'multiple_endpoint_clusterID' as a random effect, a factor with 215 levels, referring to each unique experimental comparison), but here we perform a sensitivity analysis where we select only one effect size from each of the 215 experimental comparisons.

Rather than subjectively choosing which effect size to keep for each experimental comparison, we randomly choose them (the subjective approach would require some ranking, for example, keeping any yield mass measure first if it is available, then if yield mass is not available, keeping any seed number measure.. but this would be subject to our subjective decision on what to keep).

```
# the function modified from "https://itchyshin.github.io/publication_bias/"

# function for randomly selecting 1 effect size from each experimental comparison:
```

```
choosing_es1 <- function(sim = 1){

  # splitting dataframe into the 215 experimental comparisons
  study_list <- split(data2, data2$multiple_endpoint_clusterID)

  # randomly extracting one effect size per experimental comparison
  new_dat <- map_dfr(study_list, function(x)
    x[sample(1:nrow(x), 1),c("lncvr.yi","lncvr.vi", "study_id", "info_id")])

  # running the model on the dataframe now each experimental comparison only has a single effect size
  # we no longer need multiple_endpoint_clusterID as a random effect in the model
  model <- rma.mv(yi  = lncvr.yi, V = lncvr.vi,
               random = list(~1| study_id, ~1| info_id),
               data=new_dat,
               sparse = TRUE)

  # creating a dataframe with results from a meta-analysis
  df <- data.frame(sim, beta = as.numeric(model$b))
  return(df)
}

# applying the function 1000 times
#sim1<- map_dfr(1:1000, choosing_es1)
# saving data to save time
#saveRDS(sim1,file = here("Rdata","sim1.rds"))

# load the data
sim1 <- readRDS(here("Rdata","sim1.rds"))

# percentile (bootstrap confidence interval)
quantile(sim1$beta, c(0.025, 0.975))
```

```
##       2.5%      97.5%
## -0.4885099 -0.3571283
```

```
## pdf
##   2
```

VR0 - Random effects models lnVR - absolute stability

For completeness, we also run the random effects model for lnVR (log variance ratio) which does not correct for mean-variance relationship. As above, our first model has no moderators, but has random effects for study, multiple outcome cluster, and residual

```
nullmod.vr <- rma.mv(yi=lnvr.yi,
    V=V.d2.shared.lnVR,
    random=list(~1|study_id,~1|multiple_endpoint_clusterID,~1|info_id),
    data=data2,method="ML", sparse = TRUE)
summary(nullmod.vr)
```

Re-run above model with REML for reporting in manuscript

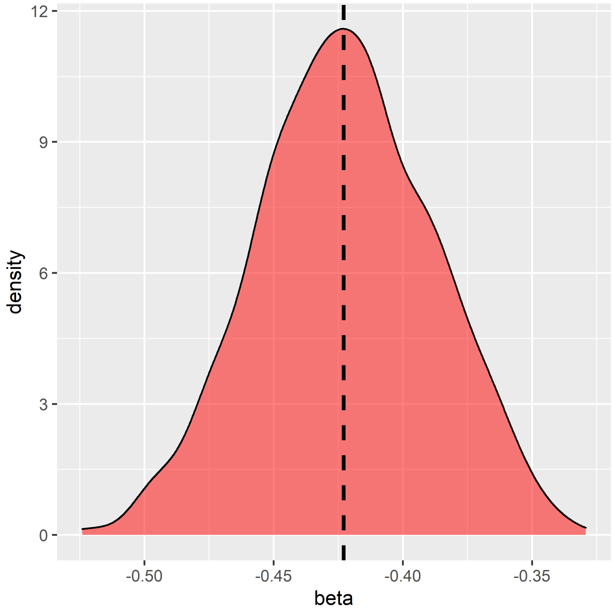

Figure S3: Sensitivity analysis on multiple outcomes per experimental comparison

```r
# null model with REML
nullmod.vr.reml <- rma.mv(yi=lnvr.yi,
    V=V.d2.shared.lnVR,
    random=list(~1|study_id,~1|multiple_endpoint_clusterID,~1|info_id),
    data=data2,method="REML", sparse = TRUE)
summary(nullmod.vr.reml)
```

```
##
## Multivariate Meta-Analysis Model (k = 498; method: REML)
##
##    logLik   Deviance        AIC        BIC       AICc
## -517.7205  1035.4410  1043.4410  1060.2753  1043.5223
##
## Variance Components:
##
##             estim    sqrt  nlvls  fixed                       factor
## sigma^2.1  0.2829  0.5319     47     no                     study_id
## sigma^2.2  0.0977  0.3126    215     no  multiple_endpoint_clusterID
## sigma^2.3  0.0485  0.2202    498     no                      info_id
##
## Test for Heterogeneity:
## Q(df = 497) = 1470.0481, p-val < .0001
##
## Model Results:
##
## estimate      se    zval    pval   ci.lb   ci.ub
##   0.3356  0.0942  3.5622  0.0004  0.1509  0.5202  ***
##
## ---
## Signif. codes:  0 '***' 0.001 '**' 0.01 '*' 0.05 '.' 0.1 ' ' 1
```

Get estimates from the REML model for reporting in the manuscript. Because pollination reduces absolute stability, we can multiply by minus 1 to change the sign and determine to what extent pollination increases variability

```
1 - exp(nullmod.vr.reml$b[[1]]*-1)
```

```
## [1] 0.2850882
```

RR0 - Random effects models lnRR - pollination benefit

We use the lnRR, log response ratio, to assess the benefit of pollination to (mean) yield across our studies

```
nullmod.rr <- rma.mv(yi=lnrr.yi,
    V=V.d2.shared.lnRR,
    random=list(~1|study_id,~1|multiple_endpoint_clusterID,~1|info_id),
    data=data2,method="ML", sparse = TRUE)
```

```
nullmod.rr.reml <- rma.mv(yi=lnrr.yi,
    V=V.d2.shared.lnRR,
    random=list(~1|study_id,~1|multiple_endpoint_clusterID,~1|info_id),
    data=data2,method="REML", sparse = TRUE)
summary(nullmod.rr.reml)
```

```
##
## Multivariate Meta-Analysis Model (k = 498; method: REML)
##
##    logLik   Deviance        AIC        BIC       AICc
## -277.0383   554.0767   562.0767   578.9110   562.1580
##
## Variance Components:
##
##              estim    sqrt  nlvls  fixed                       factor
## sigma^2.1   0.4541  0.6739     47     no                     study_id
## sigma^2.2   0.0564  0.2375    215     no  multiple_endpoint_clusterID
## sigma^2.3   0.0605  0.2461    498     no                      info_id
##
## Test for Heterogeneity:
## Q(df = 497) = 10194.2387, p-val < .0001
##
## Model Results:
##
## estimate      se    zval    pval   ci.lb   ci.ub
##   0.7144  0.1072  6.6653  <.0001  0.5044  0.9245  ***
##
## ---
## Signif. codes:  0 '***' 0.001 '**' 0.01 '*' 0.05 '.' 0.1 ' ' 1
```

Calculating how much yield increases with pollination, with confidence intervals

```
exp(nullmod.rr.reml$b[[1]])-1 # 104%
nullmod.rr.reml.res <- (orchaRd::mod_results(nullmod.rr.reml, mod = "Int"))
exp(nullmod.rr.reml.res$mod_table$lowerCL)-1
exp(nullmod.rr.reml.res$mod_table$upperCL)-1
```

```
## [1] 1.04305
## [1] 0.6559213
## [1] 1.520682
```

SD0 - lnSD (arm-based) analyses

The arm-based model we run is equivalent to a random effects model using lnCVR, though here we are directly estimating (and correcting for) the slope for how log(SD) changes with log(mean).

In the model output, *treatmentpollinated* is the difference between the estimated intercepts for excluded plants and pollinated plants, it is the equivalent as the estimate from our random effects model using lnCVR. The arm-based estimate is -0.265, qualitatively similar to the lnCVR estimate of -0.379. This is because overall, at 0.949, log(mean) has a near 1:1 relationship with log(SD) as is assumed when we use lnCVR. As with lnCVR, we can convert the difference in intercepts to measure the *percentage change in stability* by using 1-exp(*treatmentpollinated*). Our overall estimate from the overall arm-based model is that pollinated plants are 23.3% less variable than excluded plants.

Rather than manually producing and specifying the VCV matrix for shared control comparisons as we did above, we use the robust() function to construct a robust estimate of the VCV matrix.

```
mod02 <- rma.mv(yi=lnsd,
    V=vlnsd,
    mod = ~ 1 + treatment + scale(lnm, scale = FALSE),
    random=list(~1|study_id,~1|multiple_endpoint_clusterID, ~1|info_id, ~1|obs),
    data=data_long, sparse=TRUE)
summary(mod02)

#setting cluster to be shared control cluster
mod02r <- robust(mod02, cluster = data_long$sharedcontrol_clusterID)

# % change
1-exp(mod02r$b[[2]])

# lower CI
1-exp(mod02r$ci.lb[[2]])

# upper CI
1-exp(mod02r$ci.ub[[2]])
```

```
##
## Multivariate Meta-Analysis Model (k = 996; method: REML)
##
##    logLik   Deviance        AIC        BIC       AICc
## -959.4186  1918.8373  1932.8373  1967.1424  1932.9510
##
## Variance Components:
##
##               estim    sqrt  nlvls  fixed                       factor
## sigma^2.1    0.4299  0.6557     47     no                     study_id
## sigma^2.2    0.0000  0.0000    215     no  multiple_endpoint_clusterID
## sigma^2.3    0.1409  0.3753    498     no                      info_id
## sigma^2.4    0.1061  0.3257    996     no                          obs
##
## Test for Residual Heterogeneity:
```

```
## QE(df = 993) = 7528.5698, p-val < .0001
##
## Test of Moderators (coefficients 2:3):
## QM(df = 2) = 4070.0346, p-val < .0001
##
## Model Results:
##
##                            estimate      se     zval    pval    ci.lb    ci.ub
## intrcpt                      1.5235  0.1044  14.5930  <.0001   1.3189   1.7282
## treatmentpollinated         -0.2656  0.0312  -8.5153  <.0001  -0.3268  -0.2045
## scale(lnm, scale = FALSE)    0.9485  0.0149  63.4546  <.0001   0.9192   0.9778
##
## intrcpt                    ***
## treatmentpollinated        ***
## scale(lnm, scale = FALSE)  ***
##
## ---
## Signif. codes:  0 '***' 0.001 '**' 0.01 '*' 0.05 '.' 0.1 ' ' 1
##
## [1] 0.2332848
## [1] 0.3056627
## [1] 0.1533622
```

Multilevel meta-analysis models with moderators

lnCVR models - effect of moderators on relative stability

CVR2 - lnCVR - scale

The effect sizes have been aggregated at different scales depending on the study design - different numbers averaged to produce the mean and SD - in some studies, the experimental unit was a site, so SD indicates between-site variability (where plants may have very different growing conditions), in others, units were plants, so SD in that case indicates between-plant variability (where plants are likely to have more similar growing conditions).

```
table(data2$scale.var)
```

```
##
## cohort flower  plant   plot   site
##     79      6    161    183     69
```

In terms of scale, we think that cohorts and plots could be considered as equivalent, so we group them, but do note that cohorts represent temporal stability *within* years.

Run model with scale as a moderator

```
scalemod.V <- rma.mv(yi=lncvr.yi,
    V=V.d2.shared.lnCVR,
    mods=~scale.var-1,
    random=list(~1|study_id,~1|multiple_endpoint_clusterID,~1|info_id),
    data=data2,method="ML", sparse = TRUE)
summary(scalemod.V)
```

Likelihood ratio test to determine whether there are significant differences between scales

```
anova(nullmod,scalemod.V)
```

```
##
##         df      AIC      BIC     AICc    logLik     LRT   pval       QE
## Full     7 907.4281 936.9023 907.6566 -446.7140                690.7090
## Reduced  4 920.0963 936.9387 920.1775 -456.0482 18.6683 0.0003 772.7755
```

Run scale model with REML for manuscript

```
scalemod.V.reml <- rma.mv(yi=lncvr.yi,
    V=V.d2.shared.lnCVR,
     mods=~scale.var-1,
    random=list(~1|study_id,~1|multiple_endpoint_clusterID,~1|info_id),
    data=data2,method="REML", sparse = TRUE)
summary(scalemod.V.reml)
```

```
##
## Multivariate Meta-Analysis Model (k = 498; method: REML)
##
##    logLik   Deviance        AIC        BIC       AICc
## -443.4438   886.8877   900.8877   930.3054   901.1181
##
## Variance Components:
##
##             estim    sqrt  nlvls  fixed                       factor
## sigma^2.1  0.0511  0.2260     47     no                     study_id
## sigma^2.2  0.0062  0.0788    215     no  multiple_endpoint_clusterID
## sigma^2.3  0.0424  0.2059    498     no                      info_id
##
## Test for Residual Heterogeneity:
## QE(df = 494) = 690.7090, p-val < .0001
##
## Test of Moderators (coefficients 1:4):
## QM(df = 4) = 66.7022, p-val < .0001
##
## Model Results:
##
##                       estimate      se     zval    pval    ci.lb    ci.ub
## scale.varflower        -1.1426  0.3049  -3.7470  0.0002  -1.7402  -0.5449  ***
## scale.varplant         -0.4745  0.0767  -6.1886  <.0001  -0.6248  -0.3242  ***
## scale.varcohort/plot   -0.1646  0.0731  -2.2502  0.0244  -0.3079  -0.0212    *
## scale.varsite          -0.4348  0.1092  -3.9801  <.0001  -0.6489  -0.2207  ***
##
## ---
## Signif. codes:  0 '***' 0.001 '**' 0.01 '*' 0.05 '.' 0.1 ' ' 1
```

Calculate R2

```
##   R2_marginal R2_coditional
##     0.2338402     0.6741647
```

Calculate site level effect for manuscript

```
1 - exp(scalemod.V.reml$b[[4]])
```

```
## [1] 0.3526106
```

CVR1 - lnCVR - crops

The effect sizes come from three different crops, here we test whether the crops have different average effects

```
cropmod.V <- rma.mv(yi=lncvr.yi,
    V=V.d2.shared.lnCVR,
    mods=~crop-1,
    random=list(~1|study_id,~1|multiple_endpoint_clusterID,~1|info_id),
    data=data2,method="ML", sparse = TRUE)
summary(cropmod.V) # Vf, p=0.143
```

Likelihood ratio test to determine whether there are significant differences between crops species

```
anova(cropmod.V,nullmod) # p=0.0026
```

```
##
##          df      AIC      BIC     AICc    logLik    LRT   pval       QE
## Full      6 914.6395 939.9031 914.8106 -451.3198               721.1368
## Reduced   4 920.0963 936.9387 920.1775 -456.0482 9.4568 0.0088 772.7755
```

Run crop model with REML for manuscript

```
cropmod.V.reml <- rma.mv(yi=lncvr.yi,
    V=V.d2.shared.lnCVR,
    mods=~crop-1,
    random=list(~1|study_id,~1|multiple_endpoint_clusterID,~1|info_id),
    data=data2,method="REML", sparse = TRUE)
summary(cropmod.V.reml)
```

```
##
## Multivariate Meta-Analysis Model (k = 498; method: REML)
##
##    logLik   Deviance        AIC        BIC       AICc
## -447.8900   895.7800   907.7800   933.0074   907.9521
##
## Variance Components:
##
##             estim    sqrt  nlvls  fixed                       factor
## sigma^2.1  0.0697  0.2639     47     no                     study_id
## sigma^2.2  0.0097  0.0984    215     no  multiple_endpoint_clusterID
## sigma^2.3  0.0423  0.2056    498     no                      info_id
##
## Test for Residual Heterogeneity:
## QE(df = 495) = 721.1368, p-val < .0001
##
```

```
## Test of Moderators (coefficients 1:3):
## QM(df = 3) = 49.7913, p-val < .0001
##
## Model Results:
##
##                     estimate      se     zval    pval    ci.lb    ci.ub
## cropApple            -0.8309  0.1691  -4.9130  <.0001  -1.1624  -0.4994  ***
## cropFaba bean        -0.2121  0.1042  -2.0353  0.0418  -0.4163  -0.0078    *
## cropOilseed rape     -0.3629  0.0760  -4.7758  <.0001  -0.5119  -0.2140  ***
##
## ---
## Signif. codes:  0 '***' 0.001 '**' 0.01 '*' 0.05 '.' 0.1 ' ' 1
```

Get estimates for the manuscript

```
1 - exp(cropmod.V.reml$b[[1]]) # apple
1 - exp(cropmod.V.reml$b[[2]]) # faba bean
1 - exp(cropmod.V.reml$b[[3]]) # osr
```

```
## [1] 0.5643522
## [1] 0.1910837
## [1] 0.304375
```

Calculate R2

```
##   R2_marginal R2_coditional
##     0.2014821     0.7224395
```

CVR5 & CVR6 - lnCVR - pollination treatment type

We would hypothesise that there are differences between animal-pollination treatments, for example, we expect that an open pollination treatment will be more variable, because there is uncertainty whether the pollinators are present, particularly in comparison to treatments where pollination is maximised.

Run the moderator model

```
pollmod.V <- rma.mv(yi=lncvr.yi,
    V=V.d2.shared.lnCVR,
    mods=~poll-1,
    random=list(~1|study_id,~1|multiple_endpoint_clusterID,~1|info_id),
    data=data2,method="ML", sparse = TRUE)
summary(pollmod.V)
```

Likelihood ratio test to determine whether there are significant differences between crop pollination treatments.

```
anova(pollmod.V,nullmod)
```

```
##
##          df      AIC      BIC     AICc    logLik    LRT   pval       QE
## Full      9 925.5102 963.4056 925.8791 -453.7551              757.0545
## Reduced   4 920.0963 936.9387 920.1775 -456.0482 4.5861 0.4684 772.7755
```

Run the pollinator moderator model with REML and print model summary.

```
pollmod.V.reml <- rma.mv(yi=lncvr.yi,
    V=V.d2.shared.lnCVR,
    mods=~poll-1,
    random=list(~1|study_id,~1|multiple_endpoint_clusterID,~1|info_id),
    data=data2,method="REML", sparse = TRUE)
summary(pollmod.V.reml)
```

```
##
## Multivariate Meta-Analysis Model (k = 498; method: REML)
##
##    logLik   Deviance        AIC        BIC       AICc
## -450.1280   900.2560   918.2560   956.0423   918.6294
##
## Variance Components:
##
##             estim    sqrt  nlvls  fixed                       factor
## sigma^2.1  0.0871  0.2951     47     no                     study_id
## sigma^2.2  0.0101  0.1005    215     no  multiple_endpoint_clusterID
## sigma^2.3  0.0443  0.2106    498     no                      info_id
##
## Test for Residual Heterogeneity:
## QE(df = 492) = 757.0545, p-val < .0001
##
## Test of Moderators (coefficients 1:6):
## QM(df = 6) = 40.6118, p-val < .0001
##
## Model Results:
##
##               estimate      se     zval    pval    ci.lb    ci.ub
## pollbumble     -0.3763  0.1077  -3.4923  0.0005  -0.5874  -0.1651  ***
## pollhand       -0.4329  0.0889  -4.8689  <.0001  -0.6072  -0.2586  ***
## pollhoneybee   -0.2564  0.1321  -1.9413  0.0522  -0.5153   0.0025    .
## pollhoverfly   -0.0884  0.1746  -0.5061  0.6128  -0.4306   0.2538
## pollopen       -0.3891  0.0695  -5.5974  <.0001  -0.5253  -0.2528  ***
## pollsolitary   -0.2248  0.1655  -1.3588  0.1742  -0.5491   0.0995
##
## ---
## Signif. codes:  0 '***' 0.001 '**' 0.01 '*' 0.05 '.' 0.1 ' ' 1
```

Run a different moderator model, this time for us to test whether open pollination treatment is different from the others - we first simplify pollination treatment to two groups, 'open' and 'other'

```
data2$poll2 <- data2$poll
levels(data2$poll2) <- list("other"=c("bumble","hand","honeybee","hoverfly","solitary"), "open"="open")
```

Run the moderator model

```
poll2mod.V <- rma.mv(yi=lncvr.yi,
    V=V.d2.shared.lnCVR,
    mods=~poll2-1,
```

```
    random=list(~1|study_id,~1|multiple_endpoint_clusterID,~1|info_id),
    data=data2,method="ML", sparse = TRUE)
summary(poll2mod.V) # they have similar estimates
```

```
##
## Multivariate Meta-Analysis Model (k = 498; method: ML)
##
##    logLik   Deviance        AIC        BIC       AICc
## -456.0473   651.8389   922.0946   943.1476   922.2165
##
## Variance Components:
##
##              estim    sqrt  nlvls  fixed                       factor
## sigma^2.1  0.0882  0.2969     47     no                     study_id
## sigma^2.2  0.0102  0.1008    215     no  multiple_endpoint_clusterID
## sigma^2.3  0.0424  0.2060    498     no                      info_id
##
## Test for Residual Heterogeneity:
## QE(df = 496) = 765.0545, p-val < .0001
##
## Test of Moderators (coefficients 1:2):
## QM(df = 2) = 35.9964, p-val < .0001
##
## Model Results:
##
##              estimate      se     zval    pval    ci.lb    ci.ub
## poll2other   -0.3755  0.0738  -5.0903  <.0001  -0.5200  -0.2309  ***
## poll2open    -0.3782  0.0683  -5.5360  <.0001  -0.5121  -0.2443  ***
##
## ---
## Signif. codes:  0 '***' 0.001 '**' 0.01 '*' 0.05 '.' 0.1 ' ' 1
```

Likelihood ratio test to determine whether there are significant differences between pollination tes

```
anova(pollmod.V,poll2mod.V)
```

```
##
##          df      AIC      BIC     AICc    logLik    LRT   pval       QE
## Full      9 925.5102 963.4056 925.8791 -453.7551               757.0545
## Reduced  5 922.0946 943.1476 922.2165 -456.0473 4.5843 0.3327 765.0545
```

```
anova(poll2mod.V,nullmod)
```

```
##
##          df      AIC      BIC     AICc    logLik    LRT   pval       QE
## Full      5 922.0946 943.1476 922.2165 -456.0473               765.0545
## Reduced  4 920.0963 936.9387 920.1775 -456.0482 0.0018 0.9665 772.7755
```

Re-run our model with REML for reporting in the manuscript

```
poll2mod.V.reml <- rma.mv(yi=lncvr.yi,
    V=V.d2.shared.lnCVR,
    mods=~poll2-1,
    random=list(~1|study_id,~1|multiple_endpoint_clusterID,~1|info_id),
    data=data2,method="REML", sparse = TRUE)
summary(poll2mod.V.reml)
```

```
##
## Multivariate Meta-Analysis Model (k = 498; method: REML)
##
##    logLik   Deviance        AIC        BIC       AICc
## -454.1740   908.3480   918.3480   939.3809   918.4705
##
## Variance Components:
##
##             estim    sqrt  nlvls  fixed                       factor
## sigma^2.1  0.0933  0.3054     47     no                     study_id
## sigma^2.2  0.0109  0.1043    215     no  multiple_endpoint_clusterID
## sigma^2.3  0.0426  0.2063    498     no                      info_id
##
## Test for Residual Heterogeneity:
## QE(df = 496) = 765.0545, p-val < .0001
##
## Test of Moderators (coefficients 1:2):
## QM(df = 2) = 35.0033, p-val < .0001
##
## Model Results:
##
##             estimate      se     zval    pval    ci.lb    ci.ub
## poll2other   -0.3774  0.0749  -5.0360  <.0001  -0.5243  -0.2305  ***
## poll2open    -0.3798  0.0695  -5.4656  <.0001  -0.5159  -0.2436  ***
##
## ---
## Signif. codes:  0 '***' 0.001 '**' 0.01 '*' 0.05 '.' 0.1 ' ' 1
```

Calculate R2

```
##   R2_marginal R2_coditional
##  9.508035e-06  7.098994e-01
```

CVR7 - lnCVR - pollinator activity

On the theme of different pollination treatments, we can also perform a different analysis - does more pollination increase the stability benefit? There are several ways to test this but here we can test intensity of pollination as a moderator. Several papers included an 'effort' level of pollination that we have classed as low, medium and high.

```
##
##    low medium   high
##     48      8     48
```

Make subset where poll treatment has more than one level, and remake the VCV matrix for this subset

```r
data3 <- subset(data2,poll.level!="<NA>")
V.d3.shared.lnCVR <- VCV.shared(data=data3,
    cV="cV.lnCVR",
    V = "lncvr.vi",
    cluster="sharedcontrol_clusterID",
    obs="info_id")
```

How many data points, how many publications, and which publications?

```r
length(data3$lncvr.yi)
```

```
## [1] 104
```

```r
data3$studyid<-droplevels(data3$study_id)
levels(factor(data3$study_id))
```

```
##  [1] "campbell2017"    "garratt2013"     "garratt2014"     "garratt2014AGEE"
##  [5] "garratt2018"     "hudenwenz2014"   "jauker2008"      "ladurner2004"
##  [9] "mallinger2015"   "perezmendez2020" "sabbahi2005"
```

```r
length(levels(factor(data3$study_id)))
```

```
## [1] 11
```

Run model with pollination effort level as moderator

```r
pollintensmod <- rma.mv(yi=lncvr.yi,
    V=V.d3.shared.lnCVR,
    mods=~poll.level-1,
    random=list(~1|study_id,~1|multiple_endpoint_clusterID,~1|info_id),
    data=data3,method="ML", sparse = TRUE)
```

Run corresponding null model with this subsetted dataset and VCV matrix and then perform a likelihood ratio test to determine whether there are significant differences between pollination effort levels

```r
nullmod.poll.level <- rma.mv(yi=lncvr.yi,
    V=V.d3.shared.lnCVR,
    random=list(~1|study_id,~1|multiple_endpoint_clusterID,~1|info_id),
    data=data3,method="ML", sparse = TRUE)
anova(pollintensmod,nullmod.poll.level)
```

```
##
##         df      AIC      BIC     AICc    logLik    LRT   pval       QE
## Full     6 203.3556 219.2220 204.2216 -95.6778               155.1974
## Reduced  4 200.1781 210.7557 200.5822 -96.0891 0.8225 0.6628 155.8178
```

Re-run with reml

```
pollintensmod.reml <- rma.mv(yi=lncvr.yi,
    V=V.d3.shared.lnCVR,
    mods=~poll.level-1,
    random=list(~1|study_id,~1|multiple_endpoint_clusterID,~1|info_id),
    data=data3,method="REML", sparse = TRUE)
```

lnCVR - how yield is measured

Run model with response measure as moderator; we find that the lnCVR estimate is greater for 'fertilization' measures (e.g. fruit set and seed per fruit) than for yield measures that are typically measured at a whole-plant or larger scale (e.g. number of pods, number of seeds, yield mass).

```
respmod.V <- rma.mv(yi=lncvr.yi,
    V=V.d2.shared.lnCVR,
    mods=~response-1,
    random=list(~1|study_id,~1|multiple_endpoint_clusterID,~1|info_id),
    data=data2,method="ML", sparse = TRUE)
summary(respmod.V)
```

```
##
## Multivariate Meta-Analysis Model (k = 498; method: ML)
##
##    logLik   Deviance        AIC        BIC       AICc
## -449.6090   638.9623   915.2180   948.9028   915.5124
##
## Variance Components:
##
##              estim    sqrt  nlvls  fixed                       factor
## sigma^2.1  0.0598  0.2446     47     no                     study_id
## sigma^2.2  0.0134  0.1157    215     no  multiple_endpoint_clusterID
## sigma^2.3  0.0302  0.1737    498     no                      info_id
##
## Test for Residual Heterogeneity:
## QE(df = 493) = 698.4265, p-val < .0001
##
## Test of Moderators (coefficients 1:5):
## QM(df = 5) = 57.8056, p-val < .0001
##
## Model Results:
##
##                        estimate      se     zval    pval    ci.lb    ci.ub
## responsefruitset        -0.6016  0.0893  -6.7397  <.0001  -0.7765  -0.4266  ***
## responsepodnum          -0.2247  0.0972  -2.3114  0.0208  -0.4153  -0.0342    *
## responseseednum         -0.2265  0.0949  -2.3853  0.0171  -0.4125  -0.0404    *
## responseseedperfruit    -0.4034  0.0717  -5.6279  <.0001  -0.5439  -0.2629  ***
## responseyieldmass       -0.2749  0.0709  -3.8787  0.0001  -0.4138  -0.1360  ***
##
## ---
## Signif. codes:  0 '***' 0.001 '**' 0.01 '*' 0.05 '.' 0.1 ' ' 1
```

```r
anova(respmod.V,nullmod)
```

```
##
##          df      AIC      BIC     AICc    logLik     LRT   pval       QE
## Full      8 915.2180 948.9028 915.5124 -449.6090                698.4265
## Reduced   4 920.0963 936.9387 920.1775 -456.0482 12.8784 0.0119 772.7755
```

Next, we test whether these two simple groups of response measures differ from eachother. First we make a simplified variable, then run a moderator model, then compare it to the model with 5 response types and to the null model.

```r
data2$response2 <- data2$response
levels(data2$response2) <- list("fert"=c("fruitset","seedperfruit"),
    "yield"=c("podnum","seednum","yieldmass"))

respmod2.V <- rma.mv(yi=lncvr.yi,
    V=V.d2.shared.lnCVR,
     mods=~response2-1,
    random=list(~1|study_id,~1|multiple_endpoint_clusterID,~1|info_id),
    data=data2,method="ML", sparse = TRUE)
```

Likelihood ratio test to determine whether we have lost explanatory power by simplifying the response measure variable; we haven't.

```r
anova(respmod.V,respmod2.V)
```

```
##
##          df      AIC      BIC     AICc    logLik    LRT   pval       QE
## Full      8 915.2180 948.9028 915.5124 -449.6090               698.4265
## Reduced   5 913.6720 934.7250 913.7939 -451.8360 4.4540 0.2164 719.9686
```

Likelihood ratio test to determine whether 'yield' and 'fertilization' measures are significantly different

```r
anova(nullmod,respmod2.V)
```

```
##
##          df      AIC      BIC     AICc    logLik    LRT   pval       QE
## Full      5 913.6720 934.7250 913.7939 -451.8360               719.9686
## Reduced   4 920.0963 936.9387 920.1775 -456.0482 8.4243 0.0037 772.7755
```

Re-run model with REML for reporting in the manuscript, and produce estimates for the two levels

```r
respmod2.V.reml <- rma.mv(yi=lncvr.yi,
    V=V.d2.shared.lnCVR,
    mods=~response2-1,
    random=list(~1|study_id,~1|multiple_endpoint_clusterID,~1|info_id),
    data=data2,method="REML", sparse = TRUE)
# get estimates
#fert
1 - exp(respmod2.V.reml$b[[1]])
#yield
1 - exp(respmod2.V.reml$b[[2]])
```

```
## [1] 0.3752596
## [1] 0.2363777
```

Calculate R2

```
##   R2_marginal R2_coditional
##    0.07454497    0.72216951
```

lnVR models - effect of moderators on absolute (not mean-adjusted) stability

lnVR - scale

Run moderator model with scale and perform likelihood ratio test

```
scalemod.vr <- rma.mv(yi=lnvr.yi,
    V=V.d2.shared.lnVR,
     mods=~scale.var-1,
    random=list(~1|study_id,~1|multiple_endpoint_clusterID,~1|info_id),
    data=data2,method="ML", sparse = TRUE)
anova(nullmod.vr,scalemod.vr)
```

```
##
##          df       AIC       BIC      AICc    logLik    LRT   pval        QE
## Full      7 1045.2750 1074.7492 1045.5036 -515.6375               1420.5166
## Reduced   4 1046.7535 1063.5959 1046.8347 -519.3768 7.4785 0.0581 1470.0481
```

Re-run with REML for reporting in manuscript.

```
scalemod.vr.reml <- rma.mv(yi=lnvr.yi,
    V=V.d2.shared.lnVR,
    mods=~scale.var-1,
    random=list(~1|study_id,~1|multiple_endpoint_clusterID,~1|info_id),
    data=data2,method="REML", sparse = TRUE)
summary(scalemod.vr.reml)
```

```
##
## Multivariate Meta-Analysis Model (k = 498; method: REML)
##
##    logLik   Deviance        AIC        BIC       AICc
## -510.3654  1020.7309  1034.7309  1064.1486  1034.9613
##
## Variance Components:
##
##              estim    sqrt  nlvls  fixed                       factor
## sigma^2.1   0.2451  0.4950     47     no                     study_id
## sigma^2.2   0.0990  0.3146    215     no  multiple_endpoint_clusterID
## sigma^2.3   0.0487  0.2207    498     no                      info_id
##
## Test for Residual Heterogeneity:
## QE(df = 494) = 1420.5166, p-val < .0001
##
```

```
## Test of Moderators (coefficients 1:4):
## QM(df = 4) = 21.2862, p-val = 0.0003
##
## Model Results:
##
##                        estimate      se     zval    pval    ci.lb   ci.ub
## scale.varflower         -0.7591  0.5722  -1.3266  0.1846  -1.8807  0.3625
## scale.varplant           0.2776  0.1258   2.2074  0.0273   0.0311  0.5241    *
## scale.varcohort/plot     0.2350  0.1227   1.9145  0.0556  -0.0056  0.4755    .
## scale.varsite            0.6141  0.1603   3.8320  0.0001   0.3000  0.9282  ***
##
## ---
## Signif. codes:  0 '***' 0.001 '**' 0.01 '*' 0.05 '.' 0.1 ' ' 1
```

Note there is big increase in absolute variability between sites with pollination - though it is not statistically significant as tested above

lnVR - crops

```
cropmod.vr <- rma.mv(yi=lnvr.yi,
    V=V.d2.shared.lnVR,
    mods=~crop-1,
    random=list(~1|study_id,~1|multiple_endpoint_clusterID,~1|info_id),
    data=data2,method="ML", sparse = TRUE)
# compare null model and crop species model
anova(nullmod.vr,cropmod.vr)
```

```
##
##         df       AIC       BIC      AICc     logLik     LRT   pval        QE
## Full     6 1034.2211 1059.4847 1034.3922 -511.1106               1364.6854
## Reduced  4 1046.7535 1063.5959 1046.8347 -519.3768 16.5324 0.0003 1470.0481
```

```
# run with reml to get estimates
cropmod.vr.reml <- rma.mv(yi=lnvr.yi,
    V=V.d2.shared.lnVR,
    mods=~crop-1,
    random=list(~1|study_id,~1|multiple_endpoint_clusterID,~1|info_id),
    data=data2,method="REML", sparse = TRUE)
summary(cropmod.vr.reml)
```

```
##
## Multivariate Meta-Analysis Model (k = 498; method: REML)
##
##    logLik   Deviance        AIC        BIC       AICc
## -506.8543  1013.7086  1025.7086  1050.9360  1025.8807
##
## Variance Components:
##
##              estim    sqrt  nlvls  fixed                       factor
## sigma^2.1   0.1912  0.4372     47     no                     study_id
## sigma^2.2   0.0963  0.3104    215     no  multiple_endpoint_clusterID
```

```
## sigma^2.3  0.0489  0.2211    498     no                       info_id
##
## Test for Residual Heterogeneity:
## QE(df = 495) = 1364.6854, p-val < .0001
##
## Test of Moderators (coefficients 1:3):
## QM(df = 3) = 33.8467, p-val < .0001
##
## Model Results:
##
##                     estimate      se    zval    pval    ci.lb   ci.ub
## cropApple             1.0146  0.1820  5.5736  <.0001   0.6578  1.3714  ***
## cropFaba bean         0.1792  0.1484  1.2081  0.2270  -0.1115  0.4700
## cropOilseed rape      0.1409  0.1121  1.2567  0.2089  -0.0788  0.3606
##
## ---
## Signif. codes:  0 '***' 0.001 '**' 0.01 '*' 0.05 '.' 0.1 ' ' 1
```

```
# get estimates per crop
1 - exp(cropmod.vr.reml$b[[1]]*-1)
```

```
## [1] 0.6374492
```

```
1 - exp(cropmod.vr.reml$b[[2]]*-1)
```

```
## [1] 0.1640793
```

```
1 - exp(cropmod.vr.reml$b[[3]]*-1)
```

```
## [1] 0.1314081
```

```
# save this lnVR crop model
saveRDS(cropmod.vr.reml, file = "Rdata/lnvr_cropmod.reml.rds")
```

lnVR - pollination treatment type and pollination effort

```
pollmod.vr <- rma.mv(yi=lnvr.yi,
    V=V.d2.shared.lnVR,
    mods=~poll-1,
    random=list(~1|study_id,~1|multiple_endpoint_clusterID,~1|info_id),
    data=data2,method="ML", sparse = TRUE)
# compare null model and pollinator type model
anova(nullmod.vr,pollmod.vr)
```

```
##
##         df       AIC       BIC      AICc    logLik    LRT   pval        QE
## Full     9 1053.9984 1091.8938 1054.3673 -517.9992              1457.3745
## Reduced  4 1046.7535 1063.5959 1046.8347 -519.3768 2.7551 0.7377 1470.0481
```

As discussed above, we'd expect open pollinated plants to be more variable - we test whether there is a significant difference between the open pollination treatments and the other pollination treatments

```
poll2mod.vr <- rma.mv(yi=lnvr.yi,
    V=V.d2.shared.lnVR,
    mods=~poll2-1,
    random=list(~1|study_id,~1|multiple_endpoint_clusterID,~1|info_id),
    data=data2,method="ML", sparse = TRUE)
summary(poll2mod.vr) # they have similar estimates
anova(poll2mod.vr,nullmod.vr)
```

```
##
## Multivariate Meta-Analysis Model (k = 498; method: ML)
##
##    logLik   Deviance        AIC        BIC       AICc
## -519.3328   881.9519  1048.6656  1069.7186  1048.7876
##
## Variance Components:
##
##              estim    sqrt  nlvls  fixed                       factor
## sigma^2.1   0.2784  0.5276     47     no                     study_id
## sigma^2.2   0.0969  0.3113    215     no  multiple_endpoint_clusterID
## sigma^2.3   0.0485  0.2201    498     no                      info_id
##
## Test for Residual Heterogeneity:
## QE(df = 496) = 1467.2439, p-val < .0001
##
## Test of Moderators (coefficients 1:2):
## QM(df = 2) = 12.9126, p-val = 0.0016
##
## Model Results:
##
##             estimate      se    zval    pval   ci.lb   ci.ub
## poll2other    0.3520  0.1088  3.2354  0.0012  0.1388  0.5653  **
## poll2open     0.3239  0.1009  3.2088  0.0013  0.1260  0.5217  **
##
## ---
## Signif. codes:  0 '***' 0.001 '**' 0.01 '*' 0.05 '.' 0.1 ' ' 1
##
##
##         df       AIC       BIC      AICc    logLik    LRT   pval        QE
## Full     5 1048.6656 1069.7186 1048.7876 -519.3328               1467.2439
## Reduced  4 1046.7535 1063.5959 1046.8347 -519.3768 0.0879 0.7669 1470.0481
```

Also as above, we test whether different pollination effort levels result in different absolute yield stability. First we have to remake vcv matrix for lnVR for subsetted dataset. Then we run the moderator model, and perform a likelihood ratio test.

```
V.d3.shared.lnVR <- VCV.shared(data=data3,
    cV="cV.lnVR",
    V = "lnvr.vi", cluster="sharedcontrol_clusterID", obs="info_id")
# model with pollination effort level as moderator
pollintensmod.vr <- rma.mv(yi=lnvr.yi,
```

```
    V=V.d3.shared.lnVR,
    mods=~poll.level-1,
    random=list(~1|study_id,~1|multiple_endpoint_clusterID,~1|info_id),
    data=data3,method="ML", sparse = TRUE)
# corresponding null model
nullmod.poll.level.vr <- rma.mv(yi=lnvr.yi,
    V=V.d3.shared.lnVR,
    random=list(~1|study_id,~1|multiple_endpoint_clusterID,~1|info_id),
    data=data3,method="ML", sparse = TRUE)
anova(pollintensmod.vr,nullmod.poll.level.vr)
```

```
##
##          df      AIC      BIC     AICc   logLik    LRT   pval       QE
## Full      6 206.0057 221.8721 206.8717 -97.0029               255.5724
## Reduced   4 202.3554 212.9330 202.7594 -97.1777 0.3496 0.8396 256.7012
```

lnVR - how yield is measured

```
respmod.vr <- rma.mv(yi=lnvr.yi,
    V=V.d2.shared.lnVR,
    mods=~response-1,
    random=list(~1|study_id,~1|multiple_endpoint_clusterID,~1|info_id),
    data=data2,method="ML", sparse = TRUE)
summary(respmod.vr)
anova(nullmod.vr,respmod.vr)
```

```
##
## Multivariate Meta-Analysis Model (k = 498; method: ML)
##
##    logLik   Deviance        AIC        BIC       AICc
## -517.2500   877.7863  1050.5001  1084.1849  1050.7946
##
## Variance Components:
##
##             estim    sqrt  nlvls  fixed                      factor
## sigma^2.1  0.2552  0.5052     47     no                    study_id
## sigma^2.2  0.0981  0.3132    215     no  multiple_endpoint_clusterID
## sigma^2.3  0.0469  0.2166    498     no                     info_id
##
## Test for Residual Heterogeneity:
## QE(df = 493) = 1393.5434, p-val < .0001
##
## Test of Moderators (coefficients 1:5):
## QM(df = 5) = 17.8296, p-val = 0.0032
##
## Model Results:
##
##                         estimate      se    zval    pval    ci.lb   ci.ub
## responsefruitset          0.3912  0.1127  3.4703  0.0005   0.1703  0.6122  ***
## responsepodnum            0.2233  0.1211  1.8443  0.0651  -0.0140  0.4607    .
## responseseednum           0.4066  0.1192  3.4104  0.0006   0.1729  0.6403  ***
```

```
## responseseedperfruit    0.3189  0.1018  3.1307  0.0017   0.1192  0.5185   **
## responseyieldmass       0.3155  0.1021  3.0906  0.0020   0.1154  0.5156   **
##
## ---
## Signif. codes:  0 '***' 0.001 '**' 0.01 '*' 0.05 '.' 0.1 ' ' 1
##
##
##         df       AIC       BIC      AICc    logLik    LRT   pval        QE
## Full     8 1050.5001 1084.1849 1050.7946 -517.2500               1393.5434
## Reduced  4 1046.7535 1063.5959 1046.8347 -519.3768 4.2535 0.3728 1470.0481
```

lnRR - effect of moderators on pollination dependence

lnRR - how yield is measured

```
respmod.rr <- rma.mv(yi=lnrr.yi,
    V=V.d2.shared.lnRR,
    mods=~response-1,
    random=list(~1|study_id,~1|multiple_endpoint_clusterID,~1|info_id),
    data=data2,method="ML", sparse = TRUE)
summary(respmod.rr)
anova(nullmod.rr,respmod.rr)
```

```
##
## Multivariate Meta-Analysis Model (k = 498; method: ML)
##
##    logLik   Deviance        AIC        BIC       AICc
## -265.0926  1725.8603   546.1853   579.8701   546.4797
##
## Variance Components:
##
##             estim    sqrt  nlvls  fixed                       factor
## sigma^2.1  0.4491  0.6702     47     no                     study_id
## sigma^2.2  0.0589  0.2427    215     no  multiple_endpoint_clusterID
## sigma^2.3  0.0528  0.2297    498     no                      info_id
##
## Test for Residual Heterogeneity:
## QE(df = 493) = 9780.3369, p-val < .0001
##
## Test of Moderators (coefficients 1:5):
## QM(df = 5) = 74.0340, p-val < .0001
##
## Model Results:
##
##                         estimate      se    zval    pval   ci.lb   ci.ub
## responsefruitset          0.7232  0.1130  6.3999  <.0001  0.5017  0.9446  ***
## responsepodnum            0.6025  0.1161  5.1888  <.0001  0.3749  0.8301  ***
## responseseednum           0.8824  0.1140  7.7401  <.0001  0.6590  1.1059  ***
## responseseedperfruit      0.6742  0.1091  6.1784  <.0001  0.4603  0.8880  ***
## responseyieldmass         0.7532  0.1093  6.8883  <.0001  0.5389  0.9675  ***
##
## ---
```

```
## Signif. codes:  0 '***' 0.001 '**' 0.01 '*' 0.05 '.' 0.1 ' ' 1
##
##
##         df      AIC      BIC     AICc    logLik     LRT   pval         QE
## Full     8 546.1853 579.8701 546.4797 -265.0926                 9780.3369
## Reduced  4 565.6459 582.4883 565.7270 -278.8230 27.4607 <.0001 10194.2387
```

lnSD models - effect of moderators on stability

SD1 - lnSD - crops

We run individual models on subsets of the data per crop, first we run a model for apples only.

```
mod0_apple <- rma.mv(yi=lnsd,
    V=vlnsd,
    mod = ~ 1 + treatment + scale(lnm, scale = FALSE),
    random=list(~1|study_id,~1|multiple_endpoint_clusterID,~1|info_id, ~1|obs),
    data=subset(data_long, crop == "Apple"), sparse=TRUE)
summary(mod0_apple)
```

```
##
## Multivariate Meta-Analysis Model (k = 94; method: REML)
##
##   logLik  Deviance       AIC       BIC      AICc
## -92.0027  184.0055  198.0055  215.5815  199.3549
##
## Variance Components:
##
##             estim    sqrt  nlvls  fixed                       factor
## sigma^2.1  0.3066  0.5538     12     no                     study_id
## sigma^2.2  0.0000  0.0000     32     no  multiple_endpoint_clusterID
## sigma^2.3  0.0069  0.0832     47     no                      info_id
## sigma^2.4  0.1489  0.3859     94     no                          obs
##
## Test for Residual Heterogeneity:
## QE(df = 91) = 351.5389, p-val < .0001
##
## Test of Moderators (coefficients 2:3):
## QM(df = 2) = 200.1473, p-val < .0001
##
## Model Results:
##
##                            estimate      se     zval    pval    ci.lb    ci.ub
## intrcpt                      1.0426  0.1966   5.3033  <.0001   0.6573   1.4279
## treatmentpollinated         -0.3478  0.1675  -2.0766  0.0378  -0.6760  -0.0195
## scale(lnm, scale = FALSE)    0.8059  0.0707  11.4004  <.0001   0.6674   0.9445
##
## intrcpt                    ***
## treatmentpollinated          *
## scale(lnm, scale = FALSE)  ***
##
## ---
## Signif. codes:  0 '***' 0.001 '**' 0.01 '*' 0.05 '.' 0.1 ' ' 1
```

```r
1-exp(mod0_apple$b[[2]])
```

```
## [1] 0.293725
```

Model for beans.

```r
mod0_bean <- rma.mv(yi=lnsd,
    V=vlnsd,
    mod = ~ 1 + treatment + scale(lnm, scale = FALSE),
    random=list(~1|study_id,~1|multiple_endpoint_clusterID,~1|info_id, ~1|obs),
    data=subset(data_long, crop == "Faba bean"), sparse=TRUE)
summary(mod0_bean)
```

```
##
## Multivariate Meta-Analysis Model (k = 466; method: REML)
##
##    logLik   Deviance        AIC        BIC       AICc
## -326.8483   653.6965   667.6965   696.6606   667.9427
##
## Variance Components:
##
##             estim    sqrt  nlvls  fixed                      factor
## sigma^2.1  0.7192  0.8480     15     no                    study_id
## sigma^2.2  0.1044  0.3232     94     no  multiple_endpoint_clusterID
## sigma^2.3  0.0000  0.0000    233     no                     info_id
## sigma^2.4  0.0195  0.1398    466     no                         obs
##
## Test for Residual Heterogeneity:
## QE(df = 463) = 2852.1968, p-val < .0001
##
## Test of Moderators (coefficients 2:3):
## QM(df = 2) = 4536.3857, p-val < .0001
##
## Model Results:
##
##                            estimate      se     zval    pval    ci.lb    ci.ub
## intrcpt                      1.4343  0.2301   6.2327  <.0001   0.9833   1.8853
## treatmentpollinated         -0.1530  0.0329  -4.6577  <.0001  -0.2174  -0.0886
## scale(lnm, scale = FALSE)    1.1247  0.0167  67.2596  <.0001   1.0919   1.1575
##
## intrcpt                    ***
## treatmentpollinated        ***
## scale(lnm, scale = FALSE)  ***
##
## ---
## Signif. codes:  0 '***' 0.001 '**' 0.01 '*' 0.05 '.' 0.1 ' ' 1
```

```r
1-exp(mod0_bean$b[[2]])
```

```
## [1] 0.1418981
```

Model for oilseed rape.

```
mod0_canola <- rma.mv(yi=lnsd,
    V=vlnsd,
    mod = ~ 1 + treatment + scale(lnm, scale = FALSE),
    random=list(~1|study_id,~1|multiple_endpoint_clusterID,~1|info_id, ~1|obs),
    data=subset(data_long, crop == "Oilseed rape"), sparse=TRUE)
summary(mod0_canola)
```

```
##
## Multivariate Meta-Analysis Model (k = 436; method: REML)
##
##    logLik   Deviance        AIC        BIC       AICc
## -438.6925   877.3849   891.3849   919.8801   891.6485
##
## Variance Components:
##
##             estim    sqrt  nlvls  fixed                       factor
## sigma^2.1  0.3367  0.5803     22     no                     study_id
## sigma^2.2  0.0000  0.0000    102     no  multiple_endpoint_clusterID
## sigma^2.3  0.1720  0.4147    218     no                      info_id
## sigma^2.4  0.1424  0.3774    436     no                          obs
##
## Test for Residual Heterogeneity:
## QE(df = 433) = 3501.4648, p-val < .0001
##
## Test of Moderators (coefficients 2:3):
## QM(df = 2) = 1848.2666, p-val < .0001
##
## Model Results:
##
##                             estimate      se     zval    pval    ci.lb    ci.ub
## intrcpt                       1.4705  0.1371  10.7243  <.0001   1.2017   1.7392
## treatmentpollinated          -0.3244  0.0493  -6.5860  <.0001  -0.4210  -0.2279
## scale(lnm, scale = FALSE)     0.9026  0.0210  42.8985  <.0001   0.8613   0.9438
##
## intrcpt                     ***
## treatmentpollinated         ***
## scale(lnm, scale = FALSE)   ***
##
## ---
## Signif. codes:  0 '***' 0.001 '**' 0.01 '*' 0.05 '.' 0.1 ' ' 1
```

```
1-exp(mod0_canola$b[[2]])
```

```
## [1] 0.2770707
```

SD2 - lnSD - scale

We think that a reduction in variance at the site-scale is particularly interesting, more likely to include differences in environmental conditions, so we run the lnSD model just on data from a site-scale so that we can comment on this in the main manuscript.

```r
mod1_site1 <- rma.mv(yi=lnsd,
    V=vlnsd,
    mod = ~ 1 + treatment + scale(lnm, scale = FALSE),
    random=list(~1|study_id,~1|multiple_endpoint_clusterID,~1|info_id, ~1|obs),
    data=subset(data_long, scale.var == "site"), sparse=TRUE)
summary(mod1_site1)
```

```
##
## Multivariate Meta-Analysis Model (k = 138; method: REML)
##
##    logLik   Deviance        AIC        BIC       AICc
## -150.3305   300.6610   314.6610   334.9979   315.5429
##
## Variance Components:
##
##             estim    sqrt  nlvls  fixed                      factor
## sigma^2.1  0.1296  0.3600     15     no                    study_id
## sigma^2.2  0.0000  0.0000     37     no  multiple_endpoint_clusterID
## sigma^2.3  0.2600  0.5099     69     no                     info_id
## sigma^2.4  0.1109  0.3331    138     no                         obs
##
## Test for Residual Heterogeneity:
## QE(df = 135) = 1015.0050, p-val < .0001
##
## Test of Moderators (coefficients 2:3):
## QM(df = 2) = 506.1586, p-val < .0001
##
## Model Results:
##
##                            estimate      se     zval    pval    ci.lb    ci.ub
## intrcpt                      2.0601  0.1355  15.2008  <.0001   1.7945   2.3257
## treatmentpollinated         -0.4260  0.0910  -4.6812  <.0001  -0.6043  -0.2476
## scale(lnm, scale = FALSE)    0.9308  0.0425  21.8930  <.0001   0.8475   1.0141
##
## intrcpt                    ***
## treatmentpollinated        ***
## scale(lnm, scale = FALSE)  ***
##
## ---
## Signif. codes:  0 '***' 0.001 '**' 0.01 '*' 0.05 '.' 0.1 ' ' 1
```

```r
1-exp(mod1_site1$b[[2]])
```

```
## [1] 0.3468543
```

Relationships between estimators and testing for ceiling effects

With the analyses above we already addressed whether biotic pollination increases crop yield stability, and whether this benefit changes when pollination effort is greater. Now we perform additional analyses to test whether the effect of pollination on yield stability changes with the yield benefit of pollination and the yield.

Relationships between pollination benefit and yield stability

First, we test whether effect of pollination on yield stability changes with the yield benefit of pollination.

CVR3 - Relative stability and pollination benefit; lnCVR vs lnRR

```
lncvr.lnrr.mod <- rma.mv(yi=lncvr.yi,
    V=V.d2.shared.lnCVR,
    mods=~lnrr.yi,
    random=list(~1|study_id,~1|multiple_endpoint_clusterID,~1|info_id),
    data=data2,method="ML", sparse = TRUE)
summary(lncvr.lnrr.mod)
```

```
##
## Multivariate Meta-Analysis Model (k = 498; method: ML)
##
##    logLik   Deviance        AIC        BIC       AICc
## -444.1710   628.0863   898.3420   919.3950   898.4639
##
## Variance Components:
##
##             estim    sqrt  nlvls  fixed                       factor
## sigma^2.1  0.0638  0.2525     47     no                     study_id
## sigma^2.2  0.0124  0.1113    215     no  multiple_endpoint_clusterID
## sigma^2.3  0.0259  0.1608    498     no                      info_id
##
## Test for Residual Heterogeneity:
## QE(df = 496) = 712.3687, p-val < .0001
##
## Test of Moderators (coefficient 2):
## QM(df = 1) = 26.0046, p-val < .0001
##
## Model Results:
##
##          estimate      se     zval    pval    ci.lb    ci.ub
## intrcpt   -0.2028  0.0652  -3.1084  0.0019  -0.3307  -0.0749   **
## lnrr.yi   -0.2799  0.0549  -5.0995  <.0001  -0.3874  -0.1723  ***
##
## ---
## Signif. codes:  0 '***' 0.001 '**' 0.01 '*' 0.05 '.' 0.1 ' ' 1
```

Test whether there is a significant effect of pollination benefit (lnrr.yi) on relative stability benefit (lncvr.yi). Yes, there is a significant negative relationship; as the yield benefit of pollination increases, the relative stability benefit increases.

```
anova(nullmod,lncvr.lnrr.mod)
```

```
##
##         df      AIC      BIC     AICc    logLik     LRT   pval       QE
## Full     5 898.3420 919.3950 898.4639 -444.1710               712.3687
## Reduced  4 920.0963 936.9387 920.1775 -456.0482 23.7544 <.0001 772.7755
```

Now we fit with REML for reporting in the manuscript.

```
# fit with REML
lncvr.lnrr.mod.reml <- rma.mv(yi=lncvr.yi,
    V=V.d2.shared.lnCVR,
    mods=~lnrr.yi,
    random=list(~1|study_id,~1|multiple_endpoint_clusterID,~1|info_id),
    data=data2,method="REML", sparse = TRUE)
# save model
saveRDS(lncvr.lnrr.mod.reml, file = "Rdata/lncvr_lnrr.reml.rds")
```

Absolute stability and pollination benefit; lnVR vs lnRR

```
lnvr.lnrr.mod <- rma.mv(yi=lnvr.yi,
    V=V.d2.shared.lnVR,
    mods=~lnrr.yi,
    random=list(~1|study_id,~1|multiple_endpoint_clusterID,~1|info_id),
    data=data2,method="ML", sparse = TRUE)
summary(lnvr.lnrr.mod)
```

```
##
## Multivariate Meta-Analysis Model (k = 498; method: ML)
##
##    logLik   Deviance        AIC        BIC       AICc
## -440.0386   723.3634   890.0772   911.1302   890.1992
##
## Variance Components:
##
##             estim    sqrt  nlvls  fixed                       factor
## sigma^2.1  0.1034  0.3215     47     no                     study_id
## sigma^2.2  0.0232  0.1524    215     no  multiple_endpoint_clusterID
## sigma^2.3  0.0384  0.1960    498     no                      info_id
##
## Test for Residual Heterogeneity:
## QE(df = 496) = 890.1794, p-val < .0001
##
## Test of Moderators (coefficient 2):
## QM(df = 1) = 199.0896, p-val < .0001
##
## Model Results:
##
##          estimate      se     zval    pval    ci.lb    ci.ub
## intrcpt   -0.2040  0.0728  -2.8005  0.0051  -0.3467  -0.0612   **
## lnrr.yi    0.7026  0.0498  14.1099  <.0001   0.6050   0.8002  ***
##
## ---
## Signif. codes:  0 '***' 0.001 '**' 0.01 '*' 0.05 '.' 0.1 ' ' 1
```

Test whether there is a signficant effect - yes, there is a significant postive relationship, as pollination benefit (lnrr.yi) increases, yield becomes more variable with pollination. This is expected due to the positive mean variance relationship and the positive impact of animal-pollination on yield.

```
anova(nullmod.vr,lnvr.lnrr.mod)
```

```
##
##          df       AIC       BIC      AICc    logLik      LRT   pval        QE
## Full      5  890.0772  911.1302  890.1992 -440.0386                 890.1794
## Reduced   4 1046.7535 1063.5959 1046.8347 -519.3768 158.6763 <.0001 1470.0481
```

Now we fit with REML for reporting in the manuscript.

```
lnvr.lnrr.mod.reml <- rma.mv(yi=lnvr.yi,
    V=V.d2.shared.lnVR,
    mods=~lnrr.yi,
    random=list(~1|study_id,~1|multiple_endpoint_clusterID,~1|info_id),
    data=data2,method="REML", sparse = TRUE)
# save model
saveRDS(lnvr.lnrr.mod.reml, file = "Rdata/lnvr_lnrr.reml.rds")
```

CVR4 & RR1 - Stability and mean yield

Now we test for ceiling effects. We look primarily in the yield of plants that received biotic pollination, because if pollination improves yield then these are the plants that should be closer to a yield ceiling. The ceiling effect hypothesis is that pollination pushes yield toward an upper limit, beyond which it cannot increase, so yields become more similar.

We calculate z-scores within groups to make yield values approximately comparable across response variables and crop species - we don't know the yield ceiling within each particular study context (for example, some studies may have applied high levels of fertilizer), but we can standardize yield according to crop, response metric, and scale at which results are collected.

```
data2$z.meanpoll <- ave(data2$meanpoll,
    list(data2$crop,data2$response,data2$scale.res), FUN=scale)
```

We test the association between standardized yield of pollinated plants and lnCVR.

```
# moderator model
zmeanpollmod.V <- rma.mv(yi=lncvr.yi,
    V=V.d2.shared.lnCVR,
    mods=~z.meanpoll,
    random=list(~1|study_id,~1|multiple_endpoint_clusterID,~1|info_id),
    data=data2,method="ML", sparse = TRUE)
```

```
## Warning: Rows with NAs omitted from model fitting.
```

```
# reml
zmeanpollmod.V.reml <- rma.mv(yi=lncvr.yi,
    V=V.d2.shared.lnCVR,
    mods=~z.meanpoll,
    random=list(~1|study_id,~1|multiple_endpoint_clusterID,~1|info_id),
    data=data2,method="REML", sparse = TRUE)
```

```
## Warning: Rows with NAs omitted from model fitting.
```

```
summary(zmeanpollmod.V.reml)
```

```
##
## Multivariate Meta-Analysis Model (k = 497; method: REML)
##
##    logLik   Deviance        AIC        BIC       AICc
## -446.4711   892.9421   902.9421   923.9649   903.0648
##
## Variance Components:
##
##              estim    sqrt  nlvls  fixed                       factor
## sigma^2.1   0.1019  0.3193     46     no                     study_id
## sigma^2.2   0.0091  0.0953    214     no  multiple_endpoint_clusterID
## sigma^2.3   0.0305  0.1746    497     no                      info_id
##
## Test for Residual Heterogeneity:
## QE(df = 495) = 748.0462, p-val < .0001
##
## Test of Moderators (coefficient 2):
## QM(df = 1) = 15.7892, p-val < .0001
##
## Model Results:
##
##              estimate      se     zval    pval    ci.lb    ci.ub
## intrcpt       -0.3785  0.0658  -5.7487  <.0001  -0.5075  -0.2494  ***
## z.meanpoll    -0.1297  0.0326  -3.9736  <.0001  -0.1936  -0.0657  ***
##
## ---
## Signif. codes:  0 '***' 0.001 '**' 0.01 '*' 0.05 '.' 0.1 ' ' 1
```

```
# save model
saveRDS(zmeanpollmod.V.reml, file = "Rdata/lncvr_zyield.reml.rds")
```

Repeat as above but for absolute variability using lnVR.

```
#moderator model
zmeanpollmod.vr <- rma.mv(yi=lnvr.yi,
    V=V.d2.shared.lnVR,
    mods=~z.meanpoll,
    random=list(~1|study_id,~1|multiple_endpoint_clusterID,~1|info_id),
    data=data2,method="ML", sparse = TRUE)

# reml
zmeanpollmod.V.reml <- rma.mv(yi=lnvr.yi,
    V=V.d2.shared.lnVR,
    mods=~z.meanpoll,
    random=list(~1|study_id,~1|multiple_endpoint_clusterID,~1|info_id),
    data=data2,method="REML",sparse = TRUE)
# save model
saveRDS(zmeanpollmod.V.reml, file = "Rdata/lnvr_zyield.reml.rds")
```

Finally, we look at the relationship between pollination dependence and *auto-pollinated (control)* plant yield. As above, we standardize yield per crop, response measure and scale, but do so for the yield of plants excluded from pollination this time.

Because of our shared control values, this is a bit more complicated, as when there are shared controls, there are several pollination yield benefit effect sizes for each excluded control value.

```
data2$z.meanexc <- ave(data2$meanexc,
    list(data2$crop,data2$response,data2$scale.res), FUN=scale)
data2s <- subset(data2,z.meanexc!="NaN")

# need to remove duplicate shared control values, how many are there?
levels(data2s$sharedcontrol_clusterID) # 344 shared control clusters, there are not many
data2s <- data2s %>% distinct(sharedcontrol_clusterID, .keep_all = TRUE)

# we don't need to make a shared control cluster VCV matrix, because we've removed any shared control v
```

Run moderator model. There is a significant negative slope; when the yield of excluded plants is low, then the yield benefit of pollination (pollination dependence) is greater.

```
zmeanexc.lnrr <- rma.mv(yi=lnrr.yi,
    V=lnrr.vi,
    mods=~z.meanexc,
    random=list(~1|study_id,~1|multiple_endpoint_clusterID,~1|info_id),
    data=data2s,method="ML", sparse = TRUE)
```

REML version

```
zmeanexc.lnrr.reml <- rma.mv(yi=lnrr.yi,
    V=lnrr.vi,
    mods=~z.meanexc,
    random=list(~1|study_id,~1|multiple_endpoint_clusterID,~1|info_id),
    data=data2s,method="REML", sparse = TRUE)
summary(zmeanexc.lnrr.reml)
```

```
##
## Multivariate Meta-Analysis Model (k = 343; method: REML)
##
##    logLik   Deviance        AIC        BIC       AICc
## -172.2343   344.4686   354.4686   373.6280   354.6477
##
## Variance Components:
##
##             estim    sqrt  nlvls  fixed                       factor
## sigma^2.1  0.3801  0.6165     46     no                     study_id
## sigma^2.2  0.0291  0.1705    145     no  multiple_endpoint_clusterID
## sigma^2.3  0.0579  0.2406    343     no                      info_id
##
## Test for Residual Heterogeneity:
## QE(df = 341) = 7074.8837, p-val < .0001
##
## Test of Moderators (coefficient 2):
## QM(df = 1) = 44.9397, p-val < .0001
##
## Model Results:
##
##            estimate      se     zval     pval    ci.lb    ci.ub
```

```
## intrcpt      0.6685  0.1001   6.6788  <.0001   0.4723   0.8646  ***
## z.meanexc   -0.1590  0.0237  -6.7037  <.0001  -0.2054  -0.1125  ***
##
## ---
## Signif. codes:  0 '***' 0.001 '**' 0.01 '*' 0.05 '.' 0.1 ' ' 1
```

```
# save model
saveRDS(zmeanexc.lnrr.reml, file = "Rdata/lnrr_zmeanexc.reml.rds")
```

```
## pdf
##   2
```

```
## pdf
##   2
```

Produce figure S3 to show relationship between yield of excluded plants and pollination benefit to pollinated plants for each experimental comparison.

```
## pdf
##   2
```

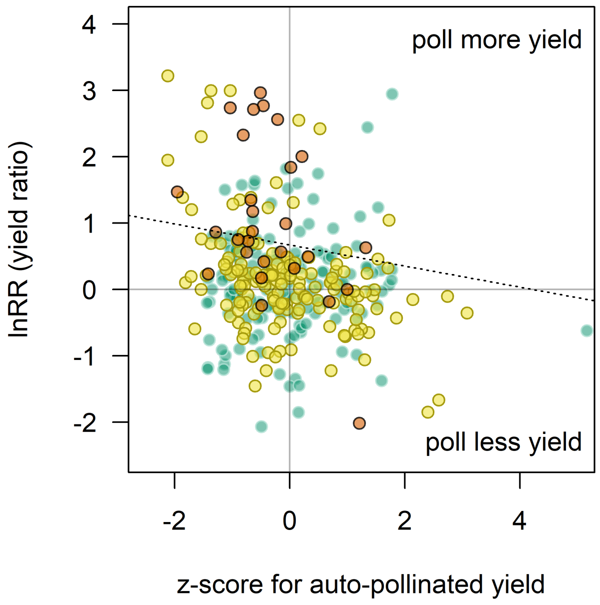

Figure S4: Yield of excluded plants vs lnRR

Sensitivity analyses

Leave-one-out sensitivity analysis for lnCVR

We use a leave-one-out sensitivity analysis but rather than leaving-out individual effect sizes and seeing how this affects our overall estimate, we leave out whole studies; in this way we are able to test whether some *studies* exerted a disproportionate effect on our overall null model estimates.

```r
data2$study_id <- as.factor(data2$study_id)

LeaveOneOut_effectsize <- list()
for(i in 1:length(levels(data2$study_id))){
    # getting VCV ready
    dat <- data2[data2$study_id != levels(data2$study_id)[i], ]
    V.d2.shared.lnCVR <- VCV.shared(data=dat,
   cV="cV.lnCVR", V = "lncvr.vi", cluster="sharedcontrol_clusterID", obs="info_id")

      # leave one out
LeaveOneOut_effectsize[[i]] <-
   rma.mv(yi = lncvr.yi, V = V.d2.shared.lnCVR,
   random = list(~1|study_id,~1|multiple_endpoint_clusterID,~1|info_id),
   method = "REML", data = data2[data2$study_id!= levels(data2$study_id)[i], ])}

  # writing function for extracting est, ci.lb, and ci.ub from all models
  est.func <- function(mod_E0){
  df <- data.frame(est = mod_E0$b, lower = mod_E0$ci.lb, upper = mod_E0$ci.ub)
  return(df)
}


#using dplyr to form data frame
MA_CVR_E_v2 <- lapply(LeaveOneOut_effectsize,
    function(x) est.func(x))%>% bind_rows %>% mutate(left_out = levels(data2$study_id))


saveRDS(MA_CVR_E_v2,file = here("Rdata", "MA_CVR_E_v2.rds"))
```

```
## pdf
##   2
```

Egger regression for lnRR

We cannot easily check for publication bias in the lnCVR analyses (because the publication bias tests use sampling error, but we use variability as the dependent variable). However for the log response ratio (lnRR) we use two versions of Egger's regression, which includes the inverse of effective sample size (`inv_effect_n`) as a moderator in either a null model (e.g. `RE0` or a full model containing all significant moderators; `year` and `response`). A significant `inv_effect_n` would indicate statistically significant funnel asymmetry after controlling for all other variables in the model. We can write `inv_effect_n` using the sample sizes from two groups (n_1 and n_2). We find no significant effect of inverse effective sample size in either model.

$$\frac{1}{\tilde{n}} = \frac{n_1 + n_2}{n_1 n_2} = \frac{1}{n_1} + \frac{1}{n_2},$$

```r
data2$inv_effect_n <- (1/ data2$npoll) + (1/ data2$nexc)

# Egger's regresion with null model
egger1 <- rma.mv(yi=lnrr.yi,
    V=V.d2.shared.lnRR,
    mods=~inv_effect_n,
```

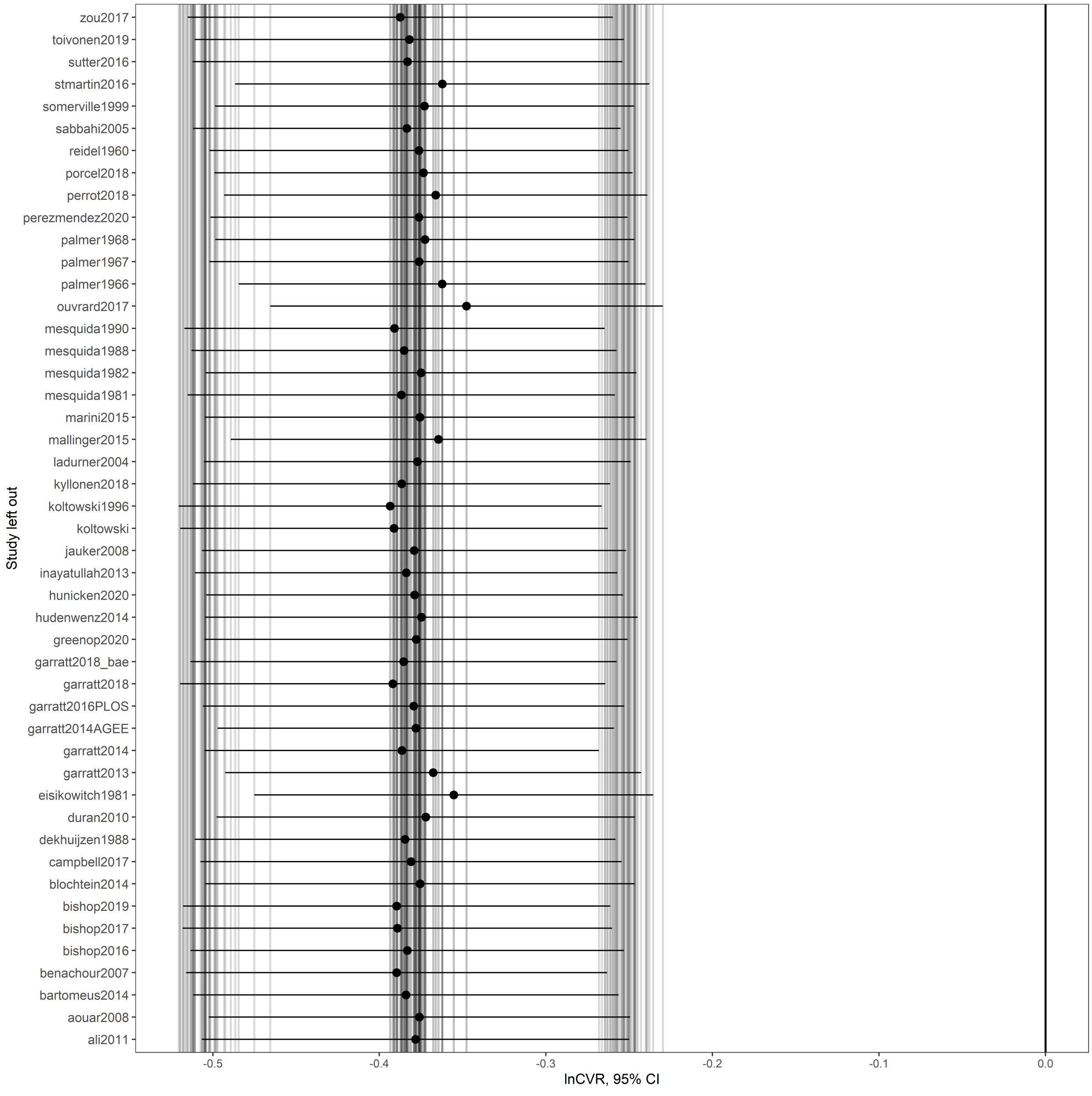

Figure S5: Leave one out analysis

```
    random=list(~1|study_id,~1|multiple_endpoint_clusterID,~1|info_id),
    data=data2,method="REML", sparse=TRUE)
summary(egger1)
```

```
##
## Multivariate Meta-Analysis Model (k = 498; method: REML)
##
##    logLik   Deviance        AIC        BIC       AICc
## -276.1279   552.2558   562.2558   583.2887   562.3782
##
## Variance Components:
##
##              estim    sqrt  nlvls  fixed                       factor
## sigma^2.1  0.4545  0.6741     47     no                     study_id
## sigma^2.2  0.0573  0.2393    215     no  multiple_endpoint_clusterID
## sigma^2.3  0.0605  0.2460    498     no                      info_id
##
## Test for Residual Heterogeneity:
## QE(df = 496) = 10187.2650, p-val < .0001
##
## Test of Moderators (coefficient 2):
## QM(df = 1) = 0.1492, p-val = 0.6993
##
## Model Results:
##
##                estimate      se     zval    pval    ci.lb   ci.ub
## intrcpt          0.7456  0.1343   5.5536  <.0001   0.4825  1.0088  ***
## inv_effect_n    -0.0828  0.2143  -0.3862  0.6993  -0.5029  0.3373
##
## ---
## Signif. codes:  0 '***' 0.001 '**' 0.01 '*' 0.05 '.' 0.1 ' ' 1
```

```
# slope -0.08 and p value for inv_effect_n is 0.7

# Eggers's regression with full model
egger2 <- rma.mv(yi=lnrr.yi,
    V=V.d2.shared.lnRR,
    mods=~inv_effect_n+year+scale.var+crop+response+poll,
    random=list(~1|study_id,~1|multiple_endpoint_clusterID,~1|info_id),
    data=data2,method="REML", sparse=TRUE)
summary(egger2)
```

```
##
## Multivariate Meta-Analysis Model (k = 498; method: REML)
##
##    logLik   Deviance        AIC        BIC       AICc
## -231.6144   463.2287   503.2287   586.7461   505.0548
##
## Variance Components:
##
##              estim    sqrt  nlvls  fixed                       factor
## sigma^2.1  0.1559  0.3948     47     no                     study_id
```

```
## sigma^2.2  0.0631  0.2513    215     no  multiple_endpoint_clusterID
## sigma^2.3  0.0535  0.2314    498     no                      info_id
##
## Test for Residual Heterogeneity:
## QE(df = 481) = 8309.3008, p-val < .0001
##
## Test of Moderators (coefficients 2:17):
## QM(df = 16) = 96.6653, p-val < .0001
##
## Model Results:
##
##                         estimate      se     zval    pval    ci.lb    ci.ub
## intrcpt                  35.6213  10.4512   3.4084  0.0007  15.1374  56.1052  ***
## inv_effect_n             -0.3554   0.2259  -1.5729  0.1157  -0.7981   0.0874
## year                     -0.0170   0.0052  -3.2795  0.0010  -0.0272  -0.0068   **
## scale.varplant            0.2340   0.4719   0.4959  0.6200  -0.6909   1.1588
## scale.varcohort/plot      0.1382   0.4740   0.2915  0.7707  -0.7908   1.0672
## scale.varsite             0.3176   0.4829   0.6578  0.5107  -0.6288   1.2641
## cropFaba bean            -1.1565   0.2213  -5.2246  <.0001  -1.5903  -0.7226  ***
## cropOilseed rape         -1.0426   0.2097  -4.9716  <.0001  -1.4536  -0.6316  ***
## responsepodnum           -0.0843   0.0705  -1.1960  0.2317  -0.2226   0.0539
## responseseednum           0.1829   0.0649   2.8189  0.0048   0.0557   0.3100   **
## responseseedperfruit     -0.0313   0.0532  -0.5893  0.5557  -0.1356   0.0729
## responseyieldmass         0.0487   0.0515   0.9452  0.3446  -0.0523   0.1497
## pollhand                  0.1143   0.1158   0.9868  0.3237  -0.1127   0.3412
## pollhoneybee             -0.0850   0.1195  -0.7112  0.4770  -0.3193   0.1493
## pollhoverfly             -0.1201   0.1423  -0.8439  0.3987  -0.3990   0.1588
## pollopen                 -0.0359   0.1081  -0.3319  0.7400  -0.2478   0.1761
## pollsolitary              0.0092   0.1440   0.0642  0.9488  -0.2731   0.2916
##
## ---
## Signif. codes:  0 '***' 0.001 '**' 0.01 '*' 0.05 '.' 0.1 ' ' 1
```

```
# slope -0.35 and p value for inv_effect_n is 0.11
```

Time lag bias test for lnCVR

To test whether findings have been changing over time, we test publication year of the studies as a moderator. There is no significant change in lnCVR over time. This is true for both the model with year only and where year is included in a full model with all significant moderators.

```
# null model
yearmod.V <- rma.mv(yi=lncvr.yi,
    V=V.d2.shared.lnCVR,
    mods=~year,random=list(~1|study_id,~1|multiple_endpoint_clusterID,~1|info_id),
    data=data2,method="ML", sparse = TRUE)
summary(yearmod.V)
```

```
##
## Multivariate Meta-Analysis Model (k = 498; method: ML)
##
##    logLik   Deviance        AIC        BIC       AICc
```

```
## -455.4441   650.6324   920.8881   941.9411   921.0101
##
## Variance Components:
##
##               estim    sqrt  nlvls  fixed                      factor
## sigma^2.1  0.0919  0.3031     47     no                    study_id
## sigma^2.2  0.0101  0.1004    215     no  multiple_endpoint_clusterID
## sigma^2.3  0.0422  0.2054    498     no                     info_id
##
## Test for Residual Heterogeneity:
## QE(df = 496) = 771.2197, p-val < .0001
##
## Test of Moderators (coefficient 2):
## QM(df = 1) = 1.2189, p-val = 0.2696
##
## Model Results:
##
##          estimate      se     zval    pval     ci.lb   ci.ub
## intrcpt  -10.8035  9.4431  -1.1441  0.2526  -29.3118  7.7047
## year       0.0052  0.0047   1.1040  0.2696   -0.0040  0.0144
##
## ---
## Signif. codes:  0 '***' 0.001 '**' 0.01 '*' 0.05 '.' 0.1 ' ' 1
```

```
anova(yearmod.V,nullmod)
```

```
##
##         df      AIC      BIC     AICc    logLik    LRT   pval       QE
## Full     5 920.8881 941.9411 921.0101 -455.4441               771.2197
## Reduced  4 920.0963 936.9387 920.1775 -456.0482 1.2082 0.2717 772.7755
```

```
# full model
yearmod.full.V <- rma.mv(yi=lncvr.yi,
    V=V.d2.shared.lnCVR,
    mods=~year+scale.var+crop+response+poll,
    random=list(~1|study_id,~1|multiple_endpoint_clusterID,~1|info_id),
    data=data2,method="ML", sparse = TRUE)
summary(yearmod.full.V)
```

```
##
## Multivariate Meta-Analysis Model (k = 498; method: ML)
##
##    logLik   Deviance        AIC        BIC       AICc
## -436.1685   612.0813   910.3370   990.3384   911.9270
##
## Variance Components:
##
##               estim    sqrt  nlvls  fixed                      factor
## sigma^2.1  0.0252  0.1587     47     no                    study_id
## sigma^2.2  0.0056  0.0748    215     no  multiple_endpoint_clusterID
## sigma^2.3  0.0322  0.1793    498     no                     info_id
##
## Test for Residual Heterogeneity:
```

```
## QE(df = 482) = 640.1133, p-val < .0001
##
## Test of Moderators (coefficients 2:16):
## QM(df = 15) = 50.7756, p-val < .0001
##
## Model Results:
##
##                       estimate      se     zval    pval     ci.lb   ci.ub
## intrcpt                -9.6047  8.0542  -1.1925  0.2331  -25.3906  6.1811
## year                    0.0039  0.0040   0.9864  0.3240   -0.0039  0.0118
## scale.varplant          0.7145  0.2754   2.5943  0.0095    0.1747  1.2542   **
## scale.varcohort/plot    0.9741  0.2820   3.4546  0.0006    0.4215  1.5268  ***
## scale.varsite           0.7729  0.2839   2.7221  0.0065    0.2164  1.3293   **
## cropFaba bean           0.3349  0.2057   1.6284  0.1034   -0.0682  0.7380
## cropOilseed rape        0.3148  0.1778   1.7707  0.0766   -0.0336  0.6631    .
## responsepodnum          0.2662  0.1235   2.1554  0.0311    0.0241  0.5084    *
## responseseednum         0.2856  0.1224   2.3330  0.0196    0.0457  0.5255    *
## responseseedperfruit    0.1187  0.0941   1.2608  0.2074   -0.0658  0.3032
## responseyieldmass       0.2370  0.0979   2.4218  0.0154    0.0452  0.4288    *
## pollhand                0.0807  0.1063   0.7588  0.4480   -0.1277  0.2891
## pollhoneybee            0.1565  0.1286   1.2163  0.2239   -0.0957  0.4086
## pollhoverfly            0.3131  0.1619   1.9336  0.0532   -0.0043  0.6305    .
## pollopen                0.0578  0.0986   0.5857  0.5581   -0.1356  0.2511
## pollsolitary            0.2472  0.1562   1.5828  0.1135   -0.0589  0.5533
##
## ---
## Signif. codes:  0 '***' 0.001 '**' 0.01 '*' 0.05 '.' 0.1 ' ' 1
```

R Session Information

```
sessionInfo()
```

```
## R version 4.1.0 (2021-05-18)
## Platform: x86_64-w64-mingw32/x64 (64-bit)
## Running under: Windows 10 x64 (build 22000)
##
## Matrix products: default
##
## locale:
## [1] LC_COLLATE=English_United Kingdom.1252
## [2] LC_CTYPE=English_United Kingdom.1252
## [3] LC_MONETARY=English_United Kingdom.1252
## [4] LC_NUMERIC=C
## [5] LC_TIME=English_United Kingdom.1252
##
## attached base packages:
## [1] stats     graphics  grDevices utils     datasets  methods   base
##
## other attached packages:
##  [1] kableExtra_1.3.4    orchaRd_0.0.0.9000  metaAidR_0.0.0.9000
##  [4] MCMCglmm_2.33       ape_5.6-2           coda_0.19-4
```

```
##  [7] here_1.0.1          corpcor_1.6.10      cowplot_1.1.1
## [10] R.rsp_0.44.0        patchwork_1.1.1     forcats_0.5.1
## [13] stringr_1.4.0       dplyr_1.0.8         purrr_0.3.4
## [16] readr_2.1.2         tidyr_1.2.0         tibble_3.1.6
## [19] ggplot2_3.3.5       tidyverse_1.3.1     devtools_2.4.3
## [22] usethis_2.1.5       metafor_3.0-2       Matrix_1.3-3
##
## loaded via a namespace (and not attached):
##  [1] nlme_3.1-152      fs_1.5.2          lubridate_1.8.0   webshot_0.5.2
##  [5] httr_1.4.2        rprojroot_2.0.2   tensorA_0.36.2    R.cache_0.15.0
##  [9] tools_4.1.0       backports_1.4.1   utf8_1.2.2        R6_2.5.1
## [13] mgcv_1.8-35       DBI_1.1.2         colorspace_2.0-3  withr_2.5.0
## [17] tidyselect_1.1.2  prettyunits_1.1.1 processx_3.5.2    compiler_4.1.0
## [21] cli_3.2.0         rvest_1.0.2       xml2_1.3.3        desc_1.4.1
## [25] labeling_0.4.2    scales_1.1.1      callr_3.7.0       systemfonts_1.0.4
## [29] digest_0.6.29     svglite_2.1.0     rmarkdown_2.13    R.utils_2.11.0
## [33] pkgconfig_2.0.3   htmltools_0.5.2   sessioninfo_1.2.2 dbplyr_2.1.1
## [37] fastmap_1.1.0     rlang_1.0.2       readxl_1.3.1      rstudioapi_0.13
## [41] farver_2.1.0      generics_0.1.2    jsonlite_1.8.0    R.oo_1.24.0
## [45] magrittr_2.0.2    Rcpp_1.0.8        munsell_0.5.0     fansi_1.0.2
## [49] lifecycle_1.0.1   R.methodsS3_1.8.1 stringi_1.7.6     yaml_2.3.5
## [53] mathjaxr_1.6-0    brio_1.1.3        pkgbuild_1.3.1    grid_4.1.0
## [57] parallel_4.1.0    crayon_1.5.0      lattice_0.20-44   splines_4.1.0
## [61] haven_2.4.3       hms_1.1.1         knitr_1.37        ps_1.6.0
## [65] pillar_1.7.0      cubature_2.0.4.2  codetools_0.2-18  pkgload_1.2.4
## [69] reprex_2.0.1      glue_1.6.2        evaluate_0.15     remotes_2.4.2
## [73] modelr_0.1.8      vctrs_0.3.8       tzdb_0.2.0        testthat_3.1.2
## [77] cellranger_1.1.0  gtable_0.3.0      assertthat_0.2.1  cachem_1.0.6
## [81] xfun_0.30         broom_0.7.12      viridisLite_0.4.0 memoise_2.0.1
## [85] ellipsis_0.3.2
```
